# *Pseudomonas aeruginosa* ExoT Induces Atypical Anoikis Apoptosis in Target Host Cells by Transforming Crk Adaptor Protein into a Cytotoxin

**DOI:** 10.1371/journal.ppat.1004934

**Published:** 2015-05-28

**Authors:** Stephen Wood, Josef Goldufsky, Sasha H. Shafikhani

**Affiliations:** 1 Department of Immunology/Microbiology, Rush University Medical Center, Chicago, Illinois, United States of America; 2 Department of Internal Medicine, Rush University Medical Center, Chicago, Illinois, United States of America; 3 Cancer Center, Rush University Medical Center, Chicago, Illinois, United States of America; Purdue University, UNITED STATES

## Abstract

Previously, we demonstrated that *Pseudomonas aeruginosa* ExoT induces potent apoptosis in host epithelial cells in a manner that primarily depends on its ADP-ribosyltransferase domain (ADPRT) activity. However, the mechanism underlying ExoT/ADPRT-induced apoptosis remains undetermined. We now report that ExoT/ADPRT disrupts focal adhesion sites, activates p38β and JNK, and interferes with integrin-mediated survival signaling; causing atypical anoikis. We show that ExoT/ADPRT-induced anoikis is mediated by the Crk adaptor protein. We found that Crk^-/-^ knockout cells are significantly more resistant to ExoT-induced apoptosis, while Crk^-/-^ cells complemented with Crk are rendered sensitive to ExoT-induced apoptosis. Moreover, a dominant negative (DN) mutant form of Crk phenocopies ExoT-induced apoptosis both kinetically and mechanistically. Crk is generally believed to be a component of focal adhesion (FA) and its role in cellular survival remains controversial in that it has been found to be either pro-survival or pro-apoptosis. Our data demonstrate that although Crk is recruited to FA sites, its function is likely not required for FA assembly or for survival *per se*. However, when modified by ExoT or by mutagenesis, it can be transformed into a cytotoxin that induces anoikis by disrupting FA sites and interfering with integrin survival signaling. To our knowledge, this is the first example whereby a bacterial toxin exerts its cytotoxicity by subverting the function of an innocuous host cellular protein and turning it against the host cell.

## Author Summary

We have previously demonstrated that ExoT is both necessary and sufficient to induce potent apoptosis in host epithelial cells in a manner that depends primarily on its ADP-ribosyltransferase (ADPRT) domain activity. However, the molecular basis underlying ExoT/ADPRT-induced apoptosis remains unknown. In this study, we demonstrate that ExoT/ADPRT by targeting the adaptor protein Crk, transforms this innocuous cellular protein into a cytotoxin that induces atypical anoikis by disrupting the focal adhesion sites which in turn interferes with the integrin-mediated pro-survival signaling.

## Introduction

*Pseudomonas aeruginosa* is a Gram-negative opportunistic pathogen that targets immunocompromised individuals and those with injured epithelia, making it one of the leading causes of nosocomial infections and the leading cause of morbidity and mortality in cystic fibrosis patients [1–3]. *P*. *aeruginosa* boasts a large arsenal of cell surface-associated and secreted virulence factors [4]. Prominent amongst them is the Type III Secretion System (T3SS) which contributes to the virulence of a large number of Gram-negative pathogens [5,6]. This conduit allows *P*. *aeruginosa* to directly translocate a set of peptide virulence factors, termed effector proteins, into the eukaryotic host cell, where they subvert host signal transduction pathways to advance *P*. *aeruginosa* infection [7]. To date, four T3SS effectors have been identified in *P*. *aeruginosa*: ExoU, ExoT, ExoS, and ExoY. ExoU is a potent phospholipase that induces necrotic cytotoxicity in eukaryotic cells [8,9]. ExoS and ExoT are homologous bifunctional proteins with an N-terminal GTPase activating protein (GAP) domain and a C-terminal ADP-ribosyltransferase (ADPRT) domain [10,11]. The GAP domains of ExoT and ExoS inhibit RhoA, Rac1, and Cdc42, small GTPases [12–15], while the ADPRT domains of ExoS and ExoT modify non-overlapping host targets. The ExoS ADPRT domain targets many host proteins including Ras, Ral, Rab, and Rac, and the ADPRT activity of ExoT modifies CrkI/II (the two isoforms of Crk) adaptor proteins and the glycolytic enzyme PGK1 [16–21]. Finally, ExoY is an adenylate cyclase that functions as an edema factor [22,23].

Unlike *exoS*, *exoU*, and *exoY* which are encoded in subsets of clinical isolates, *exoT* is present in almost all *P*. *aeruginosa* virulent clinical strains studied thus far [24,25], suggesting a more fundamental role for this virulence factor in *P*. *aeruginosa* pathogenesis. Indeed, *P*. *aeruginosa* strains defective in ExoT exhibit reduced virulence and are impaired in dissemination in mice [11,18,26]. Moreover, Balachandran et al. recently demonstrated an elegant host defense mechanism involving ubiquitin ligase Cbl-b that specifically targets ExoT, but not ExoS or ExoU, for proteasomal degradation [26]. This finding further highlights the importance of ExoT in *P*. *aeruginosa* pathogenesis and host responses to this pathogen. We and others have demonstrated that ExoT alters actin cytoskeleton, causes cell rounding, inhibits cell migration, functions as an anti-internalization factor, blocks cell division by targeting cytokinesis at multiple steps, and inhibits wound healing [12,13,18,27]. More recently, we demonstrated that ExoT is both necessary and sufficient to induce apoptosis in HeLa cells in a manner that is primarily dependent on its ADPRT domain activity [28]. However, the mechanism underlying the ExoT-induced apoptosis in epithelial cells remains unknown.

In this report, we demonstrate that ExoT-induced apoptosis is mediated by the Crk adaptor protein. Our data strongly suggest that ExoT/ADPRT activity, by ADP-ribosylating Crk, transforms this innocuous cellular protein into a cytotoxin that causes atypical anoikis by interfering with integrin-mediated survival signaling.

## Results

### ExoT/ADPRT induces atypical anoikis apoptosis

**Fig 1:**
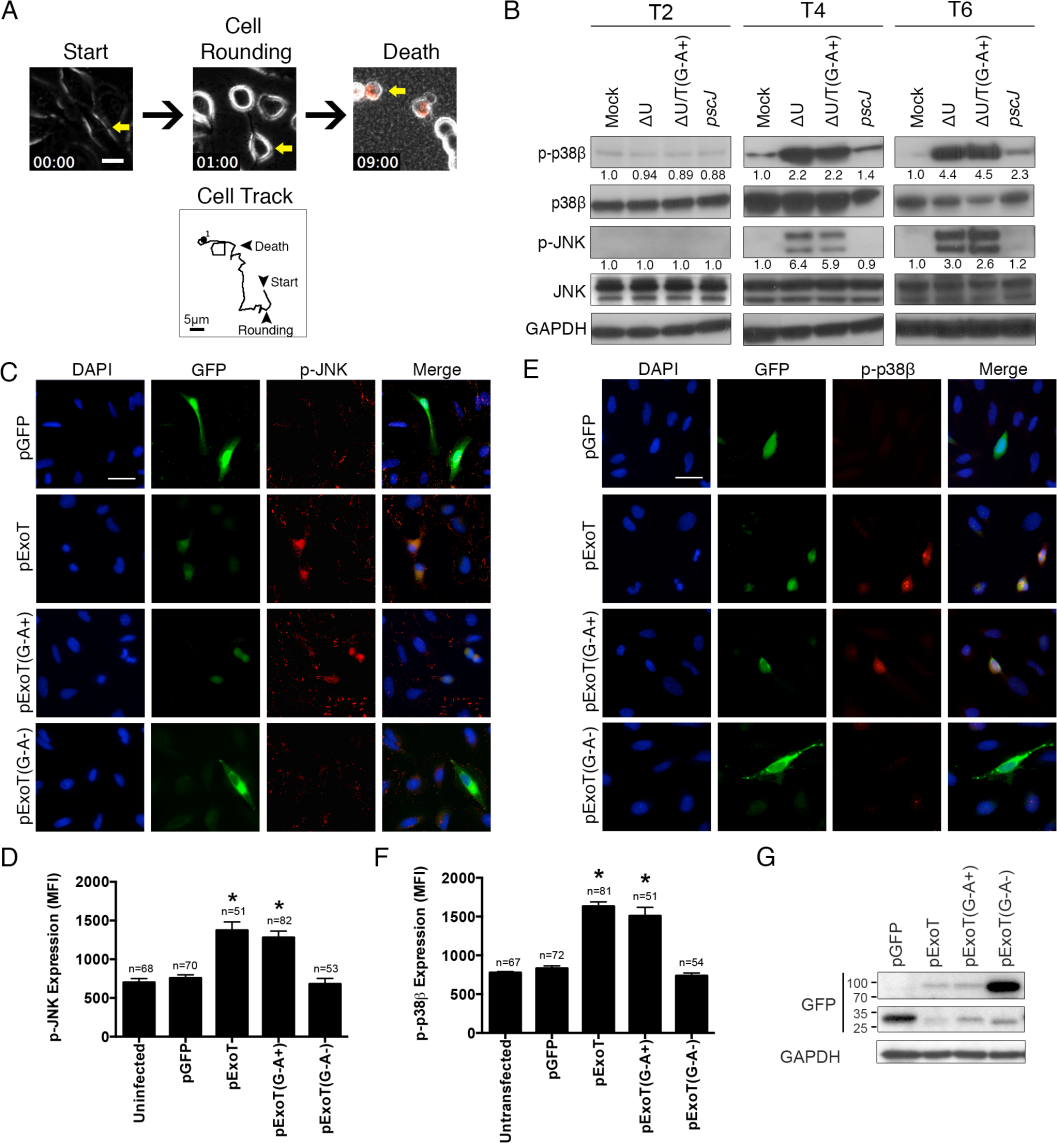
ExoT/ADPRT-intoxicated HeLa cells exhibit anoikis markers. (A) HeLa cells were infected with PA103∆*exoU* (∆U) at MOI ~10. Video images were captured every 15 min at 200× magnification. Video frames show a representative ExoT-intoxicated cell (yellow arrow) undergoing cell rounding at 1h and death, as identified by uptake of propidium iodide (red) at 9h. Cell tracking performed by ImageJ, shows this cell movement just prior to cell rounding up until cell death. (B) HeLa cells were treated with PBS (Mock) or infected with PA103∆*exoU* (∆U); PA103∆*exoU/exoT(R149K)* (∆U/T(G^-^A^+^)); or the T3SS mutant PA103 *pscJ*::*Tn5* (*pscJ*) at MOI~10. At indicated time points after infection, cell lysates were probed for phosphorylated/activated forms of p38β or JNK (p-p38β or p-JNK) by Western blotting. The fold changes in expression, as compared to mock, are shown underneath. The data were normalized to GAPDH, which was used as loading control. (C and E) HeLa cells were transfected with expression vectors harboring wild type ExoT (pExoT), ExoT with functional ADPRT domain (pExoT(G^-^A^+^)), inactive ExoT (pExoT(G^-^A^-^)), or empty vector (pGFP). ~24 hr after transfection, cells were fixed and analyzed for either phospho-JNK (p-JNK) (C) or phospho-p38β (E) by IF microscopy. Representative images are shown in (C and E) and the respective expression levels were determined by densitometry and are shown as the mean fluorescent intensity (MFI) ± SEM in (D and F) (* Signifies *p*<0.001. One-way ANOVA compared with pGFP. Scale bar = 25μm). These data indicate that ExoT/ADPRT is both necessary and sufficient to activate JNK and p38β. (G) HeLa cells transiently transfected as described above and the transient transfection efficiencies were evaluated by Western blotting using anti-GFP antibody. GAPDH was used as a loading control. All data are representative of 3 independent experiments.

Most ExoT or ExoT/ADPRT-intoxicated HeLa cells exhibited movement after cell rounding and prior to succumbing to death, as determined by the uptake of propidium iodide (PI) impermeant nuclear stain, which fluoresces red in dead or dying cells [28,29] (Fig 1A, S1 Movie). This type of cell death morphologically resembled an apoptotic programmed cell death known as anoikis, which occurs as a consequence of loss of cell adhesion and/or inappropriate cell/matrix interaction [30]. Depending on the cell line or the environmental cues, anoikis can be initiated and executed by different pathways, including the intrinsic and the extrinsic apoptotic pathways [30]. However, some common features have emerged. The common hallmarks of anoikis include: enhanced and persistent activation of p38β and JNK by phosphorylation, which is required for anoikis cell death; degradation of p130Cas and paxillin focal adhesion proteins; down activation of FAK, and down-regulation of integrin-mediated survival signaling [30–32].

We conducted a time course infection study to evaluate the possibility that ExoT/ADPRT activity induced anoikis in epithelial cells. HeLa cells were infected at a multiplicity of infection (MOI) of 10 with isogenic mutants of the PA103 strain, a clinical isolate which encodes and expresses ExoU and ExoT [24,25], including PA103∆*exoU* (∆U) which carries an in-frame deletion in the *exoU* gene but expresses ExoT; PA103∆*exoU/exoT(R149K)* (∆U/T(G^-^A^+^)) which carries an in-frame deletion in the *exoU* gene but expresses ExoT with a mutant GAP but functional ADPRT domain; or PA103 *pscJ*::*gent* ^*R*^ (T3SS mutant, unable to deliver ExoT into host cells) (S1 Table). To be able to focus on ExoT-induced cytotoxicity, we conducted these studies in the *exoU*-deleted PA103 genetic background (∆U), as we have previously described [27,28]. Moreover, because the T3SS alone induces necrotic cytotoxicity, which is completely abrogated by ExoT [28], we also left out the effectorless T3SS proficient strain (∆U∆T).

In line with our hypothesis, infection with ExoT or ExoT/ADPRT expressing *P*. *aeruginosa* strains resulted in substantial and persistent activation of both p38β and JNK by 4hr post-infection (Fig 1B). To ensure that ExoT/ADPRT activity was sufficient to activate p38β and JNK in the absence of other bacterial factors, HeLa cells were transiently transfected with pIRES2 mammalian expression vectors, harboring wild-type ExoT (pExoT), ExoT with functional ADPRT (pExoT(G^-^A^+^)), or the inactive form of ExoT (pExoT(G^-^A^-^)), all C-terminally fused to GFP, or empty vector (pGFP) (S1 Table). GFP fusion does not alter ExoT’s virulence functions [27,28]. JNK and p38β activation was evaluated by immunofluorescent (IF) microscopy of fixed cells ~24 hr post transfection. Despite reduced expression of ExoT and ExoT(G^-^A^+^) (Fig 1G) due to Cbl-b mediated proteasomal degradation of ExoT and ExoT(G^-^A^+^) [26], transient transfection with pExoT-GFP and pExoT(G^-^A^+^)-GFP resulted in significant increases in p38β and JNK activation as compared to pExoT(G^-^A^-^)-GFP or the pGFP empty vector (Fig 1C–1F), indicating that ExoT/ADPRT activity is sufficient to activate p38β and JNK.

**Fig 2:**
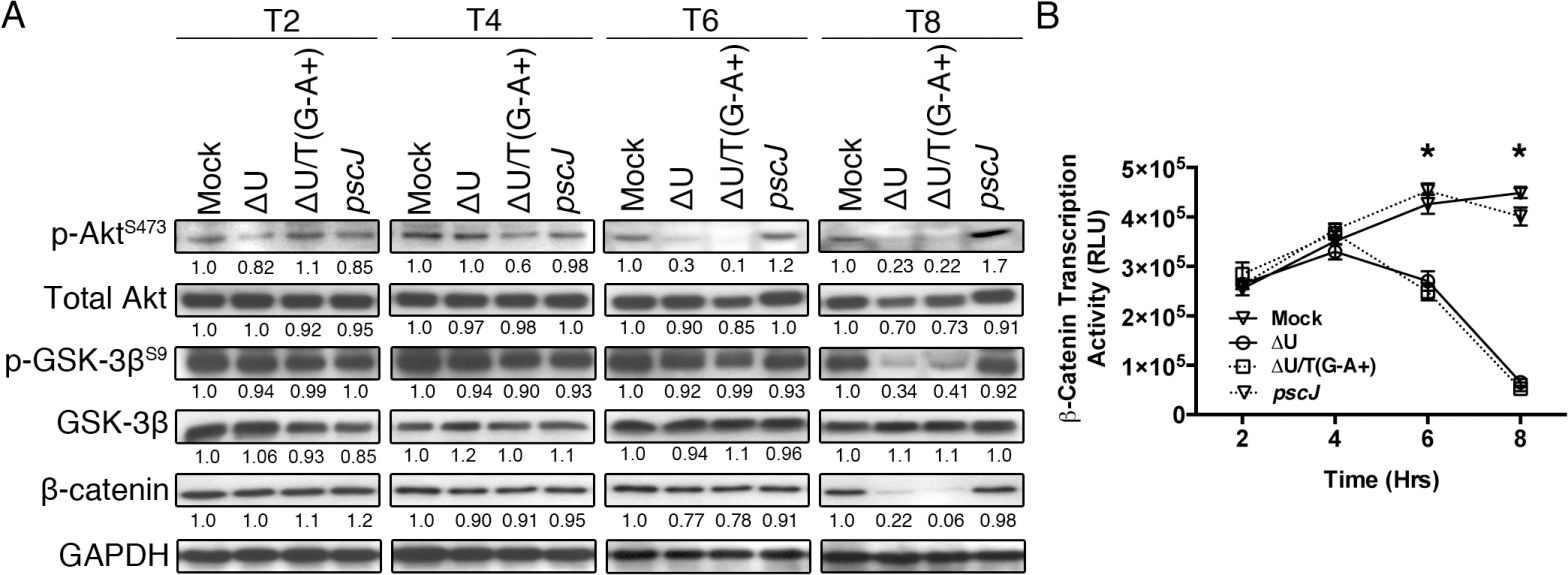
ExoT/ADPRT interferes with integrin-mediated survival signaling. (A) HeLa cells were treated with PBS or infected with PA103∆*exoU* (∆U); PA103∆*exoU/exoT(R149K)* (∆U/T(G^-^A^+^)); or the T3SS mutant PA103 *pscJ*::*Tn5* (*pscJ*) at MOI~10. At indicated time points after infection, cell lysates were probed for activated Akt (p-Akt^S473^), Akt-mediated GSK-3β inactivation by phosphorylation (p-GSK-3β^S9^), or β-catenin levels by Western blotting. The data were normalized to GAPDH and the fold changes in levels, as compared to mock, are shown underneath. (B) β-catenin transcriptional activity was assessed by transfecting HeLa cells with the TOPFlash luciferase reporter plasmid for 24 hr before infecting the cells with either ΔU, ΔU/T(G^-^A^+^), *pscJ*, or PBS. At indicated time points luciferase was assessed by a luminometer using the Luciferase Assay System (see Experimental Procedures). The experiment was performed in triplicate and the luciferase readings were normalized to baseline levels. Data are shown as mean ± SEM, * *p*<0.001, Student’s t-test).

Activation of p38β and JNK leads to disruption in integrin-mediated survival signaling, culminating in anoikis [30,31,33]. We conducted similar infection studies to evaluate the impact of ExoT/ADPRT on integrin-mediated survival signaling using Akt activation and β-catenin activity as readouts. The Akt/β-catenin pathway is an important integrin-mediated survival signaling pathway whose disruption results in anoikis [32,34–36]. Integrin interaction with extracellular matrix (ECM) leads to activation (phosphorylation) of Akt. Phospho-Akt (p-Akt) in turn activates β-catenin by blocking its inhibitor GSK-3β through phosphorylation. (Unphosphorylated GSK-3β is an inhibitor of Akt/β-catenin mediated survival signaling as it targets β-catenin for proteasomal degradation [32,34–36]). Once activated, the β-catenin transcription factor turns on the expression of pro-survival proteins [34]. In line with our hypothesis, infection with ExoT or ExoT/ADPRT-expressing strains (∆U and ∆U/T(G^-^A^+^) respectively) reduced Akt activation (p-Akt^S473^) levels by 6hr and p-Akt-mediated GSK-3β phosphorylation (inactivation) by 8hr post-infection (Fig 2A). This resulted in substantial reduction in β-catenin levels (Fig 2A) and β-catenin activity, particularly by 8hr post-infection (Fig 2B, n = 3, *p*<0.001).

**Fig 3:**
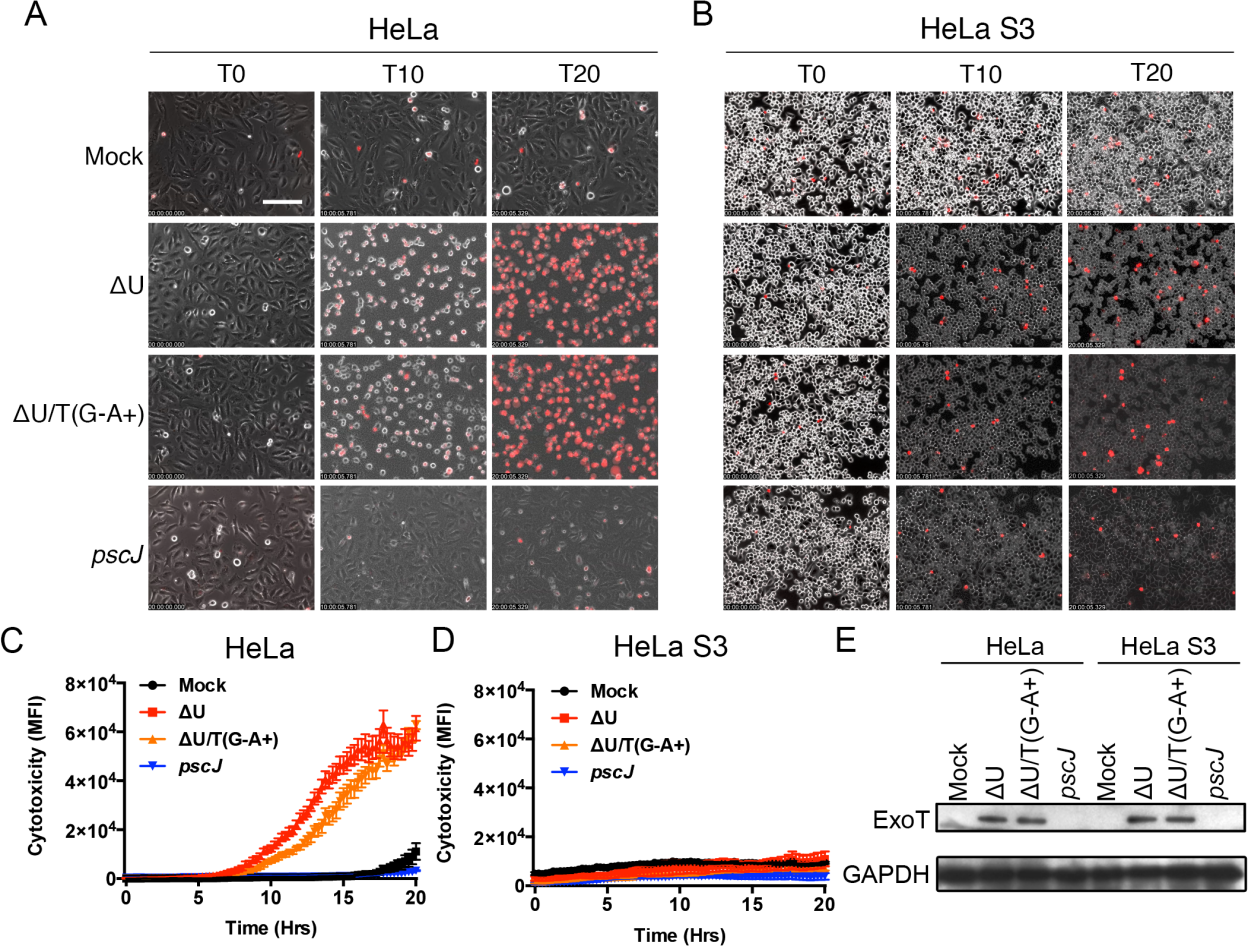
Anoikis resistant HeLa S3 cells are resistant to ExoT/ADPRT-induced cytotoxicity. HeLa or HeLa S3 cells were treated with PBS (Mock) or infected with PA103∆*exoU* (∆U); PA103∆*exoU/exoT(R149K)* (∆U/T(G^-^A^+^)); or the T3SS mutant PA103 *pscJ*::*Tn5* (*pscJ*) at MOI~10. Cytotoxicity in host cells was assessed by simultaneous phase and fluorescent time-lapse microscopy, using PI uptake (red cells are dead). Video images were captured every 15 min. selected movie frames at indicated time points are shown in (A, B) and the corresponding cytotoxicity assessment (evaluated by PI fluorescence intensity measurements for total positive pixels per frame) are shown in (C, D) respectively. Cytotoxicity is shown as the mean of 3 independent experiments. (E) ExoT level in infected HeLa and HeLa S3 cells after 4hr infection was determined by Western blotting. (Scale bar = 100 μm).

HeLa S3 cells are derived from HeLa cells through planktonic growth and are frequently used as anoikis resistant cells because their survival is adhesion independent [37,38]. We reasoned that if ExoT or ExoT/ADPRT induced anoikis in the target cells, HeLa S3 should be resistant to their cytotoxicity. To test this hypothesis, we infected HeLa and HeLa S3 cells with ExoT-expressing ∆U, ExoT/ADPRT-expressing ∆U/T(R149K), T3SS mutant *pscJ*, or PBS (Mock). Cytotoxicity was observed by time-lapse videomicroscopy and measured every 15 minutes using PI uptake as a marker for cell death [29,39]. Consistent with our previous report [28], HeLa cells were sensitive to ExoT and ExoT/ADPRT-induced cytotoxicity (Fig 3A and 3C and S2 Movie). Despite similar levels of ExoT intoxication (Fig 3E), HeLa S3 cells displayed resistance to ExoT and ExoT/ADPRT-induced cytotoxicity (Fig 3B and 3D and S3 Movie). Taken together, these data indicated that ExoT and ExoT/ADPRT induced anoikis by interfering with integrin survival signaling.

### The dominant negative mutant form of Crk phenocopies ExoT-induced apoptosis

The ADPRT domain of ExoT ADP-ribosylates a conserved arginine residue in the SH2 domain of CrkI and CrkII isoforms of Crk, disrupting Crk SH2 interactions with its cognate substrates [17,40]. Crk has been implicated in cell death, although its role in cytotoxicity remains controversial. Depending on cell type or physiological condition, Crk has been found to be either pro-apoptotic [41–45] or pro-survival [46,47]. We wished to examine the possible role of Crk in the ExoT/ADPRT-induced apoptosis in HeLa cells.

**Fig 4:**
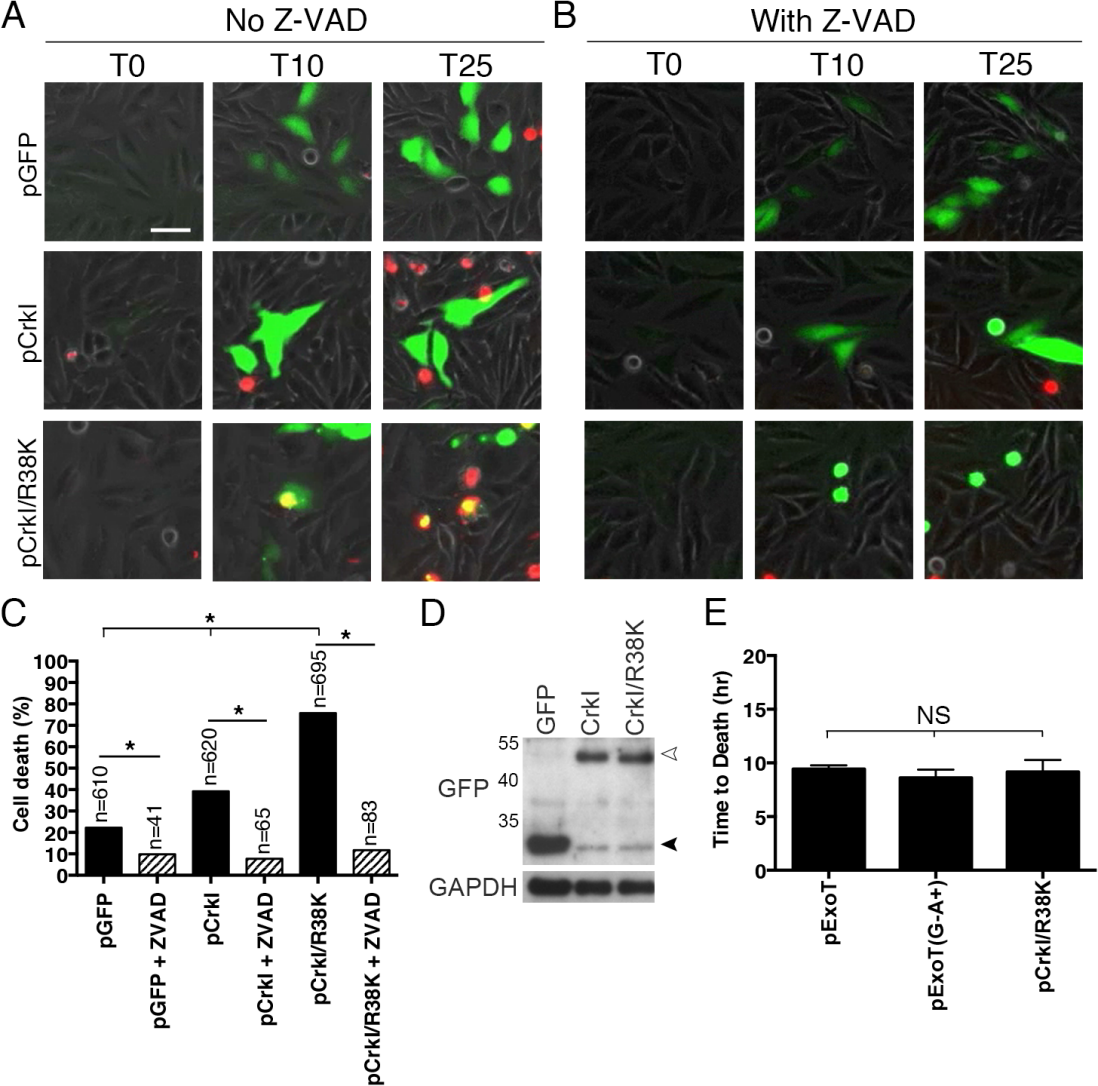
CrkI/R38K mutant phenocopies ExoT/ADPRT-induced apoptosis in HeLa cells. HeLa cells were transiently transfected with pIRES2-GFP expression vector harboring wild type CrkI (pCrkI), SH2 DN (pCrkI/R38K), all directly fused to GFP C-terminally, or empty vector (pGFP) in the absence (A) or presence (B) of Z-VAD pan-caspase inhibitor. PI was added to identify dying cells and cell death was analyzed by timelapse IF videomicroscopy. (C) The tabulated results, collected from multiple movies, are shown. (D) Transfection efficiencies were evaluated by Western blotting, using anti-GFP antibody and cells lysates from HeLa cells transfected as in (A). GAPDH was used as a loading control. The black arrowhead points to the position of GFP from the vector alone and the white arrowhead indicates CrkI-GFP and CrkI/R38K-GFP. (E) The time to death was defined as the time of expression of the transfected gene (appearance of green) to the time of PI uptake (appearance of yellow) and expressed as the mean ± SEM. Note that expression of SH2 DN CrkI induces potent apoptosis and kinetically phenocopies ExoT and ExoT/ADPRT-induced apoptosis. (* Signifies significance with *p*<0.01, χ^2^ analyses. Scale bar = 25 μm).

We transfected HeLa cells with a mammalian expression vector harboring wild type CrkI (pCrkI) or the CrkI/R38K mutant form (in which the conserved arginine 38 residue in the SH2 domain was mutated to lysine), fused at their C-termini to GFP (S1 Table), and assessed apoptosis by IF videomicroscopy in the presence of PI, as we described previously [28]. Because of low transfection efficiencies, we were unable to assess the impact of CrkII or CrkII/R38K mutation on survival in HeLa cells. Of note, CrkI/R38K has been shown to act as a DN mutant, interfering with CrkI and CrkII associated cellular activities [48,49]. Despite similar transfection efficiencies (Fig 4D), transfection with CrkI/R38K SH2 DN phenocopied ExoT and ExoT/ADPRT-induced apoptosis as it significantly increased Z-VAD sensitive apoptotic cell death in HeLa cells (Fig 4A–4C and S4 Movie; n = 610; *p*<0.001). The time to death, defined as the start time of gene expression as determined by GFP expression, to the time of death as determined by PI uptake, in the presence CrkI/R38K SH2 DN mutant was 9.1 ± 1.1 hr which is also nearly identical to the observed time to death in the presence of ExoT (8.5 ± 1.3 hr) or ADPRT (8.6 ± 0.8 hr) ([28] and Fig 4E). Similar to ExoT and ADPRT, transfection with CrkI/R38K SH2 DN also resulted in activation of JNK and p38β in HeLa cells (S1 Fig, n = 122 and n = 124 respectively, one-way ANOVA, *p*<0.001). Moreover, HeLa S3 cells were also completely resistant to CrkI/R38K SH2 DN-induced apoptosis (S2 Fig). Combined, these data indicated that ExoT-mediated apoptosis is phenocopied by the CrkI/R38K SH2 DN mutant form.

### ExoT/ADPRT transforms Crk into a cytotoxin

So far our data indicate that ExoT/ADPRT induces anoikis in a manner that likely involves Crk. The two most likely scenarios are: 1) Crk is required for survival and its modification by ExoT/ADPRT prevents it from performing its survival function; or 2) Crk is not required for survival but when it is ADP-ribosylated by ExoT/ADPRT, it is transformed into a cytotoxin which disrupts survival functions inside the cell. We favored the second scenario because; although, Crk-null (Crk^-/-^) mice die shortly after birth [50], Crk^-/-^ cells not only survive, they are actually more resistant to apoptosis [42]. These reports strongly suggest that while Crk is required for development, Crk function is not essential for cellular survival *per se*.

**Fig 5:**
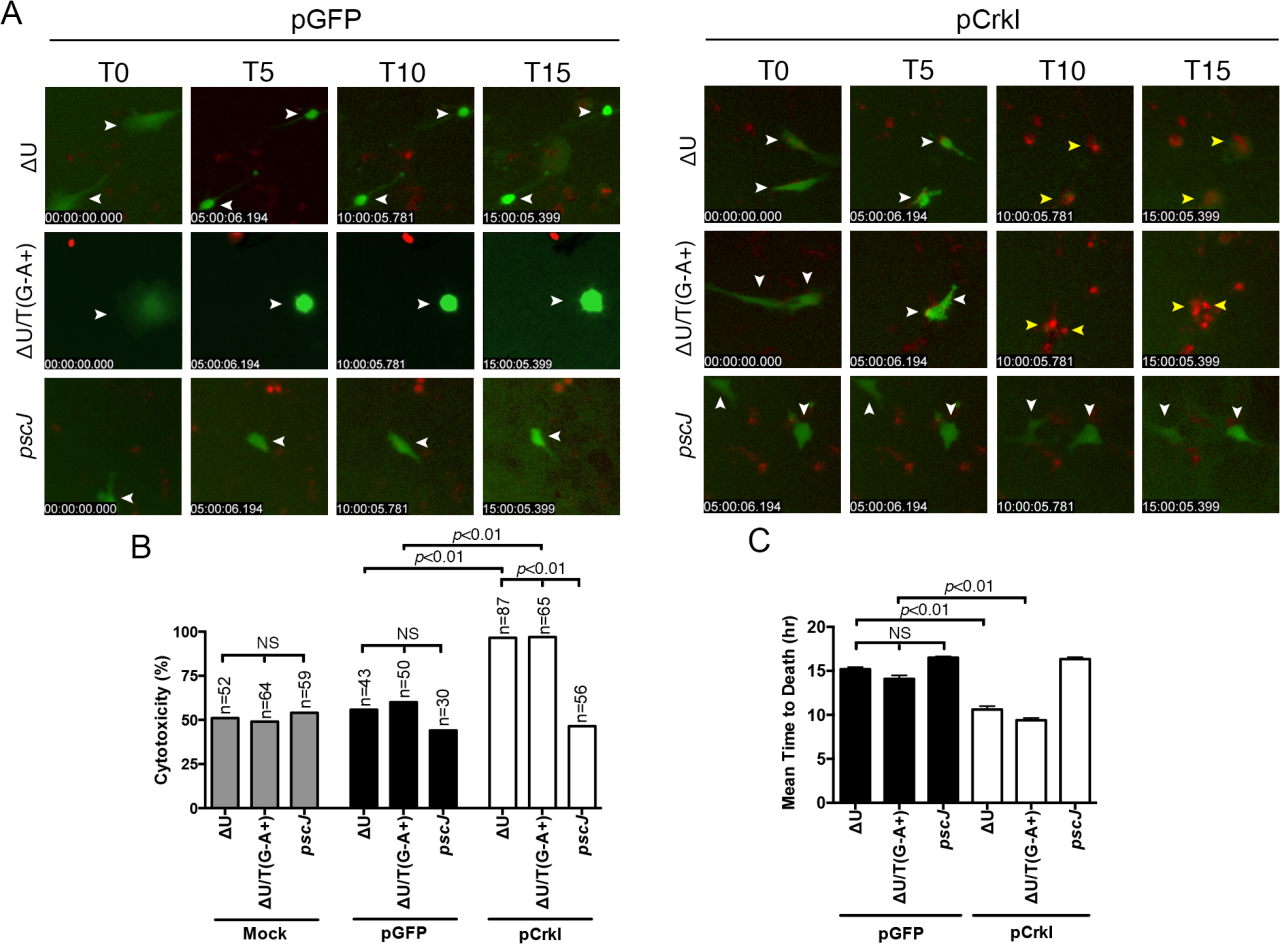
Crk mediates ExoT-induced apoptosis. Crk^-/-^ cells were transiently transfected with pCrkI, or pGFP on 3 consecutive days to obtain more transfected cells. 24 hr after the final transfection, cells were infected with either ∆U, ∆U/T(G^-^A^+^), or *pscJ* at MOI~10. Cytotoxicity of transfected host cells (green) was assessed by fluorescent time-lapse microscopy, using PI uptake (red cells are dead). Cytotoxicity is expressed as a percentage of the total number of transfected cells. Video images were captured every 15 min and selected movie frames at indicated time points are shown in (A). (For clarity, phase panels were excluded from the merged images). The corresponding tabulated data are shown in (B). (C) The corresponding time to death, defined as the time of infection to the time of PI uptake (red) is expressed as the mean ± SEM. The data in B and C comprise 3 independent experiments. Statistical analysis was performed using one-way ANOVA.

We reasoned that if ExoT or ExoT/ADPRT-induce apoptosis in epithelial cells by transforming Crk, Crk^-/-^ cells should be resistant to ExoT and ExoT/ADPRT-induced cytotoxicity. In contrast, complementing Crk^-/-^ cells with Crk should then restore their sensitivity to ExoT and ExoT/ADPRT-induced apoptosis. To address these possibilities, we complemented Crk^-/-^ mouse embryonic fibroblasts (MEFs) with either CrkI-GFP or GFP alone by transfection. The pCrkI-GFP or pGFP-transfected Crk^-/-^ cells were then infected with ExoT-expressing ∆U, ExoT/ADPRT-expressing ∆U/T(G^-^A^+^) or T3SS mutant *pscJ* and assessed for cytotoxicity by IF timelapse videomicroscopy. Although more cytotoxicity was observed in Crk^-/-^ cells that were infected with *P*. *aeruginosa*, regardless of the strain, when compared to HeLa cells, Crk^-/-^ cells complemented with CrkI-GFP were significantly more sensitive to ExoT or ExoT/ADPRT-induced cytotoxicity, as compared to GFP complemented Crk^-/-^ cells (Fig 5B and S5 and S6 Movies. Representative frames are shown in Fig 5A). Additionally, the time to death, (defined as the time of infection to the time cell death manifested by PI staining), in the presence of ∆U or ∆U/T(R149K), was also significantly faster in Crk^-/-^ cells complemented with CrkI-GFP, as compared to the GFP-complemented Crk^-/-^ cells that succumbed to death in response to infection with *P*. *aeruginosa* (Fig 5C).

**Fig 6:**
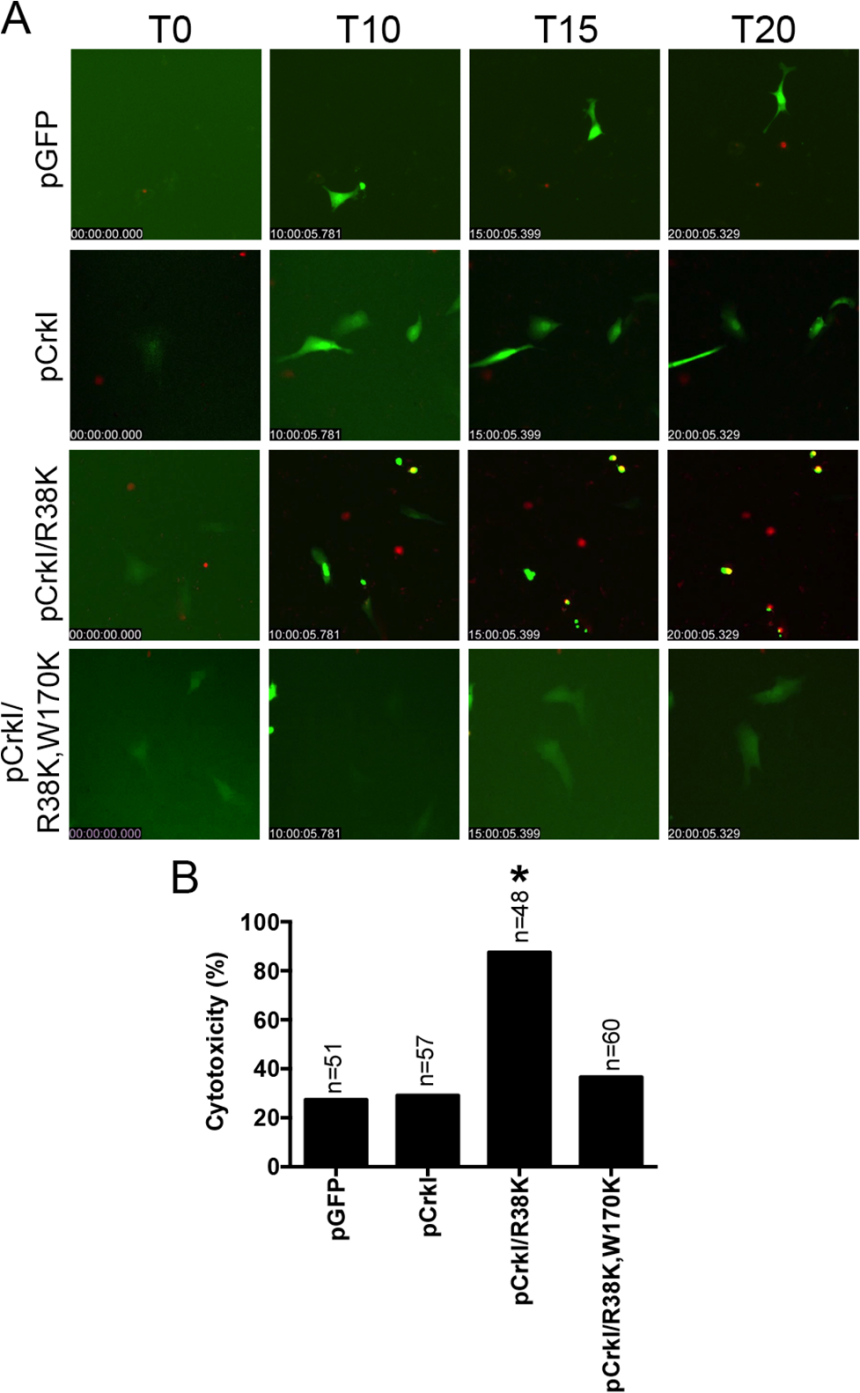
CrkI/R38K induces cytotoxicity in Crk^-/-^ cells. Crk^-/-^ cells were transiently transfected with pCrkI, pCrkI/R38K, pCrkI/R38K, W170K or the vector control pGFP. Video images were captured every 15 min and selected movie frames at indicated time points are shown in (A) (For clarity, phase panels were excluded from the merged images). (B) Cytotoxicity of transfected host cells (green) was assessed by fluorescent time-lapse microscopy, using PI uptake (red cells are dead). Cytotoxicity is expressed as a percentage of the total number of transfected cells. * Signifies significance with *p<*0.001 by χ^2^ analyses analysis. Data are representative of 3 independent experiments.

If CrkI SH2 domain modification by ExoT ADP-ribosylation or by mutagenesis (CrkI/R38K) renders CrkI into a cytotoxin, expression of CrkI/R38K SH2 mutant, but not CrkI, should also result in cytotoxicity in Crk^-/-^ cells, phenocopying ExoT’s effect in CrkI-complemented Crk^-/-^ cells. Consistent with this view, transient transfection with the CrkI/R38K SH2 mutant resulted in significantly more cytotoxicity in Crk^-/-^ cells, as compared to CrkI (Fig 6 and S7 Movie). Transfection with CrkI/R38K, W170K, which harbors null mutations in both SH2 and SH3 domains of CrkI [48], did not result in cytotoxicity in Crk^-/-^ (Fig 6), indicating that the SH3 domain of CrkI must be functional for CrkI/R38K mutant to function as a dominant negative (DN) and a cytotoxin. Corroborating this hypothesis, complementing Crk^-/-^ cells with CrkI/R38K, W170K double mutant did not render Crk^-/-^ cells sensitive to ExoT or ExoT/ADPRT-induced cell death (S3 Fig, S8 Movie), indicating that the SH3 domain of CrkI must be functional for the ADP-ribosylated CrkI to act as a cytotoxin. As expected, Crk^-/-^ cells did not express CrkI/II isoforms of Crk (S4 Fig).

### ExoT and ExoT/ADPRT, and CrkI/R38K interfere with integrin survival signaling by disrupting focal adhesion sites

We next sought to determine how ADP-ribosylation of CrkI by ExoT/ADPRT domain activity or by mutagenesis (R38K mutation) could potentially transform this cellular protein disruptive to integrin-mediated survival signaling. We hypothesized that CrkI ADP-ribosylation by ExoT/ADPRT could disrupt integrin-survival signaling by destabilizing the focal adhesion (FA) sites. We based this hypothesis on the knowledge that FAK activation and FAK/p130Cas interactions at FA sites stabilize the integrin/extracellular matrix (ECM) interactions and are required for survival signaling [51–53]. During FA assembly, integrin/ECM interaction results in activation (phosphorylation) of focal adhesion kinase (FAK), which in turn phosphorylates p130Cas and paxillin [54,55]. Phosphorylated p130Cas and paxillin then recruit Crk to FA by directly interacting with its SH2 domain [56]. Although ExoT’s impact on FA has not been investigated, Crk interactions with p130Cas and paxillin have been shown to be disrupted by ExoT/ADPRT activity [40]. Therefore, while FAK, p130Cas, and paxillin localization to FA is upstream of Crk, disruption of Crk activity by ExoT or by CrkI/R38K mutation could potentially disrupt FAK, p130Cas, and paxillin subcellular localization, and/or their maintenance, and/or their activation at FA sites. This would in turn interfere with integrin/ECM survival signaling and would lead to anoikis [53,57].

**Fig 7:**
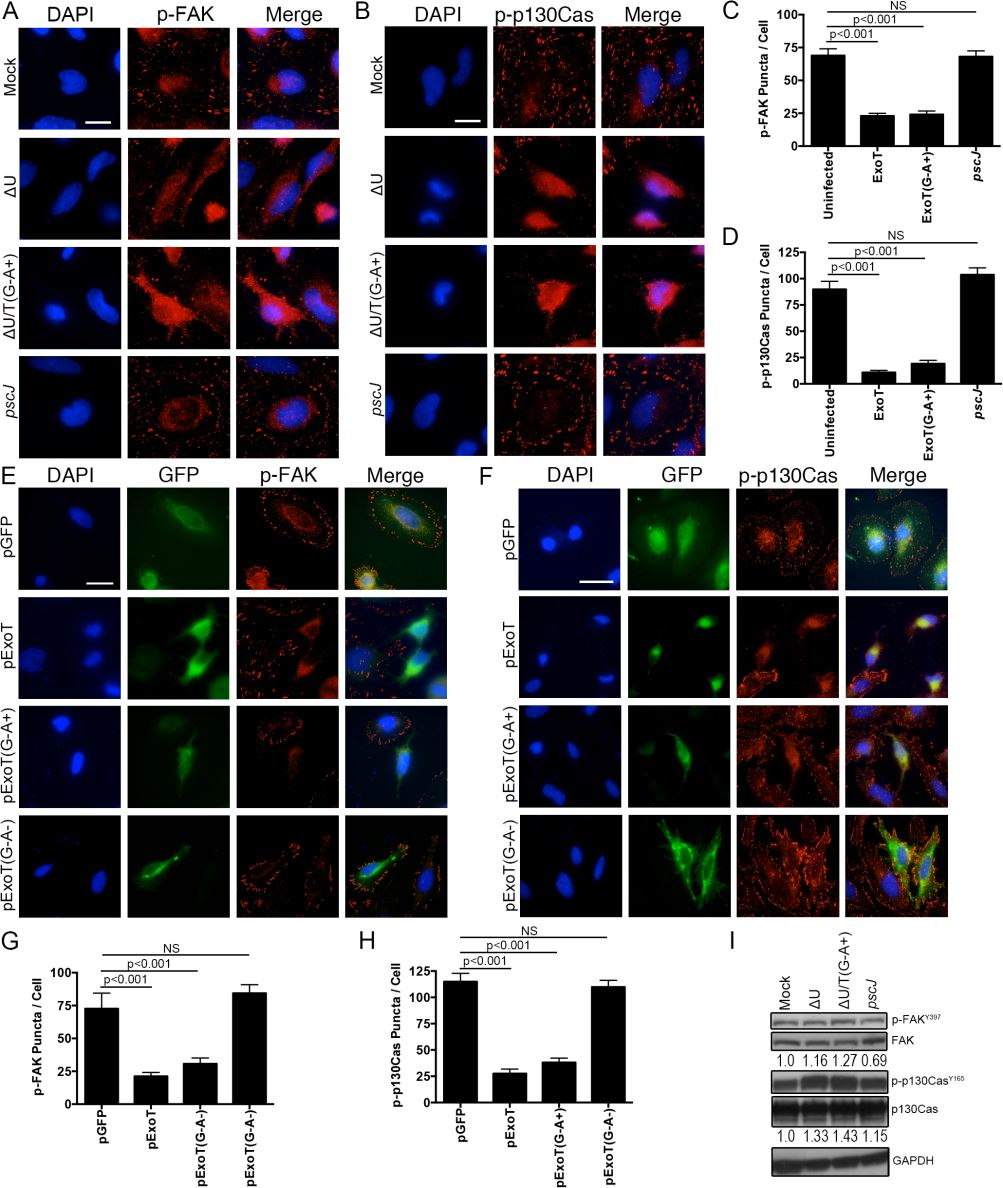
ExoT and ADPRT disrupt focal adhesion sites. (A-D) HeLa cells were treated with PBS or infected with ∆U, ∆U/T(G^-^A^+^), or *pscJ* at MOI~10. Four hours after infection, cells were fixed and stained with nuclear stain DAPI and p-FAK (A) or p-p130Cas (B). Representative images are shown in (A and B) and the tabulated results are shown in (C and D). (E-H) HeLa cells were transiently transfected with pIRES2-GFP expression vector harboring ExoT (pExoT), ExoT/ADPRT (pExoT(G^-^A^+^), or inactive ExoT (pExoT(G^-^A^-^), all C-terminally fused to GFP, or empty vector (pGFP). 24 hr after transfection, cells were fixed and stained with nuclear stain DAPI and analyzed for p-FAK and p-p130Cas localization to the FA sites. Representative images are shown in (E and F) and the tabulated results from 3 independent experiments are shown in (G and H). (Statistical analysis was performed using one-way ANOVA. Scale bar = 25 μm). (I) HeLa cells were infected as in (A). 4hr after infection, the cell lysates were probed for total FAK and p130Cas or their phosphorylated forms (p-FAK^Y397^ & p-p130Cas^Y165^ respectively) by Western blotting. Expression levels were normalized to GAPDH, which was used as the loading control.

**Fig 8:**
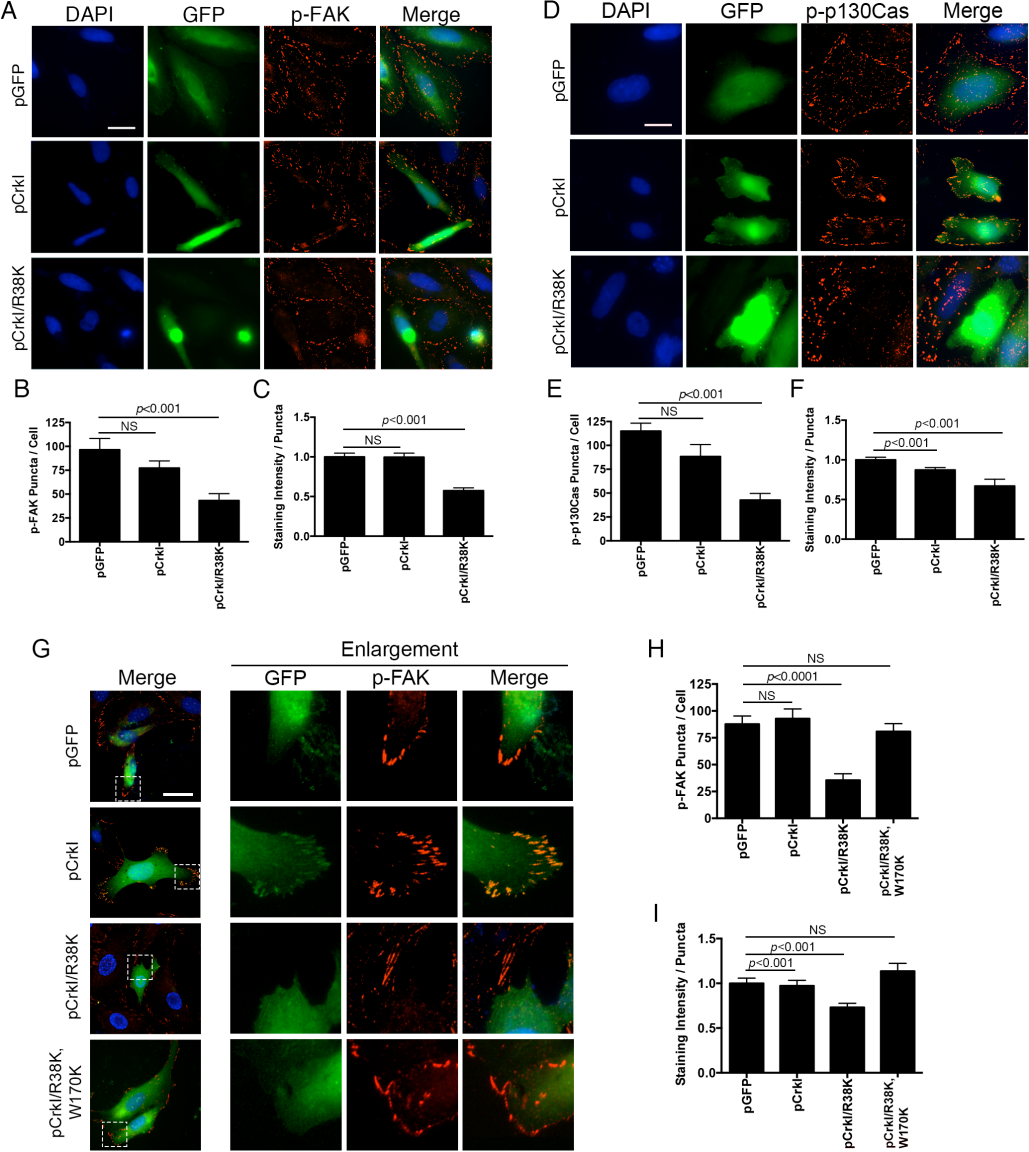
CrkI/R38K mutant disrupts FA sites in HeLa and Crk^-/-^ cells, phenocopying ExoT/ADPRT adverse effect on FA sites. HeLa cells were transiently transfected with pIRES2-GFP expression vector harboring wild-type CrkI (pCrkI), SH2 DN (pCrkI/R38K), all C-terminally fused to GFP, or empty vector (pGFP). 24 hr after transfection, cells were fixed and stained with nuclear stain DAPI and analyzed for p-FAK localization to the FA sites. Representative images are shown in (A and D) and the tabulated results from 3 independent experiments are shown in (B-C and E-F). (E) Crk^-/-^ cells were transiently transfected with pIRES2-GFP expression vector harboring wild-type CrkI (pCrkI), SH2 DN (pCrkI/R38K), or SH2 and SH3 double mutant (pCrkI/R38K,W170K), all C-terminally fused to GFP, or empty vector (pGFP). 24 hr after transfection, cells were fixed and stained with nuclear stain DAPI and analyzed for p-FAK localization to the FA sites. Representative images and enlargements of fields indicated by a white box are shown in (G) and the tabulated results from 3 independent experiments are shown in (H-I). (Statistical analysis was performed using one-way ANOVA. Scale bar = 25 μm).

To test this hypothesis, we performed the aforementioned time-course infection studies with ExoT-expressing ∆U, ExoT/ADPRT-expressing ∆U/T(G^-^A^+^), and the T3SS mutant *pscJ*, and assessed the impact of ExoT or ExoT/ADPRT on FA sites by determining the total number of FAK and p-130Cas positive FA puncta per cell and the intensity of FAK and p-130Cas stainings per FA puncta by IF microscopy (described in Methods and S5 Fig). Infection with ∆U or ∆U/T(G^-^A^+^) bacteria resulted in substantial reduction in FAK and p130Cas localization to FA sites by 4h post-infection, as manifested by reduced number of FA puncta (Fig 7A–7D) and reduced staining intensities of FAK and p130Cas per FA puncta (S6A and S6B Fig). As expected, transient transfection with expression vectors harboring ExoT (pExoT) or the ExoT/ADPRT domain (pExoT(G^-^A^+^)) also resulted in significant reduction in FAK and p130Cas localization to the FA sites (Fig 7E–7H and S6C and S6D Fig), indicating that ExoT/ADPRT activity is sufficient to disrupt FA sites. Interestingly, infection with ∆U or ∆U/T(G^-^A^+^) did not significantly affect the total cellular levels of activated (phosphorylated) FAK (p-FAK^Y397^) or activated (phosphorylated) p-130Cas (p-p130Cas^Y165^) as measured by Western blotting (Fig 7I). These data indicated that FAK and p130Cas are able to get to the FA sites where they become phosphorylated but they are not maintained at FA sites in the presence of ExoT or ExoT/ADPRT activity. Of note, in HeLa cells transfected with pCrkI-GFP, CrkI co-localized with FAK and p130Cas at FA sites (Fig 8A–8F, pCrkI panels). In contrast, CrkI/R38K did not localize to FA sites and disrupted FAK and p130Cas localization to the FA sites in HeLa cells (Fig 8A–8F, pCrkI/R38K panels), phenocopying the adverse effect of ExoT and ExoT/ADPRT on FA and supporting our hypothesis that ExoT ADP-ribosylation of CrkI renders CrkI disruptive to FA sites.

It is generally believed that Crk is an essential component of FA sites [56,58]. However, our data strongly suggested that although Crk may be recruited to FA, its function may not require for FA assembly or its dynamics. Consistent with this view, we found FAK to localize to FA sites in Crk^-/-^ cells (Fig 8G–8I), indicating that FAK subcellular localization to FA sites and its maintenance in that compartment does not require Crk in this cell line. Interestingly, in Crk^-/-^ cells that were transfected with CrkI-GFP, CrkI co-localized with FAK at FA sites (Fig 8G) indicating that CrkI can be recruited and maintained at FA sites in this cell line as well. Similar to HeLa, CrkI/R38K SH2 DN mutant did not localize to FA sites and disrupted FAK localization to FA sites in Crk^-/-^ cells that were transfected with CrkI/R38K-GFP expression vector (Fig 8G–8I). Of note, CrkI/R38K, W170K (SH2 and SH3 double mutant) did not localize to FA sites and failed to disrupt FAK localization to FA sites in Crk^-/-^ and HeLa cells (Fig 8G–8I), indicating that the SH3 domain must be functional for CrkI/R38K to act as a dominant negative.

We next asked whether CrkI presence in FA sites affected FA structures and/ or their function in in Crk^-/-^ cells. We evaluated the impact of CrkI on FA structure and function in Crk^-/-^ cells by determining how CrkI affected: (i) the number of FA puncta per cell, (ii) the ability of Crk^-/-^ cells to adhere to the surface, as determined by their surface area, and (iii) the ability of Crk^-/-^ cells to migrate, as determined by distance these cells travelled within 24 hr after transfection with CrkI. The data indicated that CrkI presence in FA sites in Crk^-/-^ cells, complemented with CrkI, did not affect their FA structures or functions (S7 Fig). Collectively, these data indicate that while CrkI can be recruited to FA, its function is not essential for FA assembly or survival. However, when modified by ExoT or by mutagenesis, it can disrupt FA sites, which would result in anoikis.

## Discussion

We have recently reported that *Pseudomonas aeruginosa* ExoT is both necessary and sufficient to induce potent apoptosis in target host cells in a manner that primarily depends on its ADPRT domain activity [28] but the mechanism underlying ExoT/ADPRT-induced apoptosis has not been determined. We now report that ExoT/ADPRT induces anoikis by disrupting FA sites. Our data demonstrate that within 4h of exposure to ExoT or ExoT/ADPRT, FA sites are destabilized (Fig 7). Concomitant with FA disruption, intoxication with ExoT or ExoT/ADPRT leads to persistent activation of p38β and JNK (Fig 1), which are known drivers of anoikis [33,59]. This in turn leads to dampened integrin-mediated survival signaling by 6–8 hr post-exposure, as manifested by reduced Akt activation and β-catenin-mediated survival signaling (Fig 2). ExoT/ADPRT-induced anoikis is atypical in that we did not observe degradation of p130Cas or down-activation of FAK (Fig 7I), both of which have been reported to occur during anoikis and be required for cytotoxicity [30–32,53,60]. Instead, ExoT or ExoT/ADPRT interfered with FAK and p130Cas subcellular localization to the FA sites (Fig 7A–7H). Our data suggest that FA sites may serve as cellular survival centers. Consistent with this notion, JNK localization to FA sites and its interaction with FAK and p130Cas in that compartment has been shown to be required for survival [57]. Of note, ExoT and ExoT/ADPRT also disrupted JNK localization at FA sites (S8 Fig and Fig 1C, compare JNK staining at FA sites in the untransfected and pGFP- transfected cells to the pExoT or pExoT(G^-^A^+^)-transfected cells which lack JNK at FA sites).

Crk is generally believed to be an essential component of FA [56,58], although it is not clear whether it is CrkI or CrkII or both that function in FA. Moreover, Crk has also been implicated in cellular survival and/or apoptosis but Crk’s role in these processes remain controversial in that it is found to be either pro-apoptotic [41–45] or pro-survival [46,47]. Our data strongly suggest that Crk function may not be essential for FA or for cellular survival *per se*, but when modified by ExoT or by mutagenesis, Crk can be transformed into a cytotoxin that interferes with survival signaling by disrupting FA (Figs 5, 6 and 8). How does modification of CrkI SH2 domain by ExoT/ADPRT-mediated ADP-ribosylation render CrkI disruptive to integrin-mediated survival signaling? We propose that ADP-ribosylation by ExoT transforms CrkI into a dominant negative mutant that is disruptive to FA structures. Our reasoning is that while these ADP-ribosylated CrkI and CrkI/R38K are unable to interact through the SH2 domain with their cognate substrates such as p130Cas and paxillin [40], they would be able to interact with a number of essential FA components, such as C3G [61,62] through the SH3 domain. This prevents their localization to FA sites, and destabilizes FA structures, thus culminating in anoikis as our data demonstrate (Figs 7 and 8). Consistent with this hypothesis, CrkI/(R38K, W170K) which harbors mutations in both SH2 and SH3 domains failed to disrupt FA sites (Fig 8), or induce apoptosis in Crk^-/-^ cells (Fig 6), or renders Crk^-/-^ cells sensitive to ExoT or ExoT/ADPRT cytotoxicity (S3 Fig, S8 Movie).

Crk has been shown to function in various host defenses against bacterial pathogens, such as inhibiting EPEC-induced actin-based pedestal formation, increasing bacterial clearance through phagocytosis of IgG-opsonized pathogens by Fcγ receptors, and potentially enhancing innate immune activation through pattern recognition receptors (PRR) by sequestering bacterial virulence factors, such as EPEC’s Tir, which interfere with PRR signaling [63–65]. Therefore, it is not surprising that pathogens have evolved mechanisms to target this central host protein in order to advance their own agenda. For example, EPEC induces phosphorylation of the major regulatory tyrosine in CrkII, preventing CrkII from sequestering Tir, thus freeing this virulence factor to promote pedestal formation [63]. Additionally, *P*. *aeruginosa* blocks host cell proliferation by inhibiting Crk’s essential function for cytokinesis [27].

In summary, we propose that ExoT by ADP-ribosylating Crk transforms this cellular protein into a cytotoxin, which induces anoikis by disrupting FA structures and interfering with the integrin survival signaling.

## Materials and Methods

### Cell culture and reagents

HeLa (ATCC), HeLa S3 (ATCC), and Crk^-/-^ cells were cultured in complete DMEM (Life Technologies) containing phenol red supplemented with 10% FCS, 1% penicillin/streptomycin, and 1% L-glutamine at 37°C in the presence of 5% CO_2_. For transfection experiments, 0.4μg plasmid DNA was used with Effectene (Qiagen) according to the manufactures protocol. Antibodies were from Cell Signaling Technologies (CST) unless otherwise noted: pAKT (#9271); Crk (BD 610036); GAPDH (GenScript A00191); pJNK (#4668); JNK (#9252); p130Cas (BD 610271); p-p130Cas(Y165) (#4015); p38β (#9212); p-p38β (#9215); FAK (BD 610087); pFAK(Y397) (#3283).

### Cytotoxicity measurement by time-lapse videomicroscopy was performed as we described previously [27,28]

Briefly, HeLa, HeLa S3, and Crk^-/-^ cells were grown in DMEM without phenol red with (for transfection studies) or without antibiotics (for infection studies) for 24 hr. These cells were then transfected with indicated expression vectors or infected with indicated strains as described [28]. 1h after transfection or at time of infection, cells were given 7μg/ml propidium iodide (Sigma) and then placed on an AxioVert Z1 microscope (Zeiss) fitted with a live-imaging culture box (Pecon) maintaining 37°C in the presence of 5% CO_2_. Time-lapse videos were taken using AxioVision 4.2.8 software. Video analysis was performed with ImageJ 1.47 software (NIH).

### Western blot

Samples were assessed by Western blot as described previously [29,66]. Briefly, cells were lysed following infection with 1% TX-100 containing a protease inhibitor cocktail (Roche Diagnostics), 100mM PMSF, and 100mM Na_3_VO_4_. Lysates were mixed with 4X SDS loading buffer and loaded onto 10% SDS-polyacrylamide gels. After resolving, gels were transferred to PVDF membranes, blocked with 5% milk, and probed overnight with primary antibody at 4°C. After washing, blots were probed with HRP-conjugated secondary antibody (Cell Signaling Technologies). Blots were developed with ECL or ECL+ reagent (GE Healthcare). Films were developed with an autoprocessor.

### Bacteria strains and plasmids

All bacterial strains and expression vectors and their sources are indicated in S1 Table. These isogenic strains were in PA103 genetic background. For infection studies, bacteria were cultured overnight in Luria-Bertani (LB) broth at 37°C without shaking. Bacteria were added at M.O.I of 10.

### Luciferase reporter assay

β-catenin transcriptional activity assay was performed as previously described [67]. HeLa cells were transfected with 400ng TOPFlash plasmid (Upstate) using Effectene (Qiagen). After 24 hr, the cells were washed with PBS and given fresh media either with our without antibiotics. The cells were either mock infected (PBS) or infected with the aforementioned strains which were resuspended in PBS. Luciferase levels were measured according to the manufacturers protocol using the Luciferase Assay System (Promega) using a Moonlight 2010 Luminometer (BD Bioscience) and normalizing to *Renilla* luciferase’s baseline luminance. Experiments were performed in triplicate for each time point.

### Immunofluorescent microscopy

Immunofluorescent microscopy was carried out as previously described [28,66,68]. Briefly, coverslips were treated with poly-l-lysine and 40μg/ml human fibronectin (Millipore) before seeding cells. After 24 hr, cells were either transfected for 17h or infected for 4h. At each end-point, cells were fixed with 10% ice-cold TCA for 10min. Cells were permeablized with 0.2% Triton X-100 (Sigma) for 15min at RT, blocked with 3% FCS for 1H at 37°C before staining overnight with primary antibody. Next, cells were washed 3x with PBS before staining with conjugated secondary antibody, AF594 or AF488 (Life Technologies) for 1hr at 37°C. The coverslip was mounted on DAPI containing VectaMount (Vector Laboratories). Cells were imaged AxioVision 4.2.8 software using an AxioVert Z1 microscope (Zeiss) using a 63X objective.

### Focal adhesion analysis

Immunofluorescent microscopy images were processed using ImageJ v1.47 (NIH). Cell outlines were first saved using the selection manager. A background subtraction was applied to the channel containing the focal adhesion marker. Next, a threshold level was applied to that channel followed by particle analysis to identify the number of puncta per cell selection and staining intensity per puncta. Each image set was processed using equal threshold and particle analysis values.

### Statistical analysis

Two-tailed Student *t*-tests, one-way ANOVA with Tukey post-hoc test, and Chi-squared analyses were used to assess significance with *p*<0.05 considered significant. Analysis was carried out with Prism 6.0 (GraphPad).

## Supporting Information

S1 Fig CrkI/R38K expression leads to activation of JNK and p38β in HeLa cells, phenocopying ExoT/ADPRT. HeLa cells were transiently transfected with pIRES2-GFP expression vector harboring wild-type CrkI (pCrkI), SH2 DN (pCrkI/R38K), or empty vector (pGFP). 24 hr after transfection, cells were fixed and analyzed for p-JNK or p-p38β by IF microscopy. Representative images are shown in (A) and (C) and the expression levels were determined by densitometry and are shown as the mean fluorescent intensity (MFI) ± SEM in (B) and (D) respectively. (* Signifies *p*<0.05 and ** signifies *p*<0.001 by one-way ANOVA. Scale bar = 25μm).
(TIF)

S2 Fig HeLa S3 cells are resistant to ExoT, ExoT/ADPRT, and CrkI/R38K mediated cytotoxicity. HeLa S3 cells were transiently transfected with pIRES2-GFP expression vector harboring ExoT (pExoT), ExoT/ADPRT (pExoT(G^-^A^+^), inactive ExoT (pExoT(G^-^A^-^), wild-type CrkI (pCrkI), or pCrkI/R38K mutant, all C-terminally fused to GFP, or empty vector (pGFP). Cytotoxicity of transfected host cells (green) was assessed by fluorescent time-lapse microscopy, using PI uptake, as the marker for cell death (red cells are dead). Video images were captured every 15 min and selected movie frames at indicated time points are shown in (A) and the corresponding data, expressed as a percentage of the total number of transfected cells, are shown in (B). Data was assessed by one-way ANOVA.
(TIF)

S3 Fig The SH3 domain of CrkI must be functional for CrkI to mediate ExoT cytotoxicity. Crk^-/-^ cells were transfected with the pCrkI/R38K,W170K-GFP vector, harboring null mutations in the SH2 and SH3 domains of CrkI, on consecutive days to increase transfection efficiency. 24 hr after final transfection, transfected cells were infected with PA103Δ*exoU* (ΔU), PA103∆*exoU/exoT(R149K)* (∆U/T(G^-^A^+^)), or the T3SS mutant PA103 *pscJ*::*Tn5* (*pscJ*) at MOI ~10. Cytotoxicity of transfected host cells (green) was assessed by fluorescent time-lapse microscopy, using PI uptake, as the marker for cell death (red cells are dead). Video images were captured every 15 min and selected movie frames at indicated time points are shown in (A) and the corresponding data, expressed as a percentage of the total number of transfected cells, are shown in (B). For clarity, phase panels were excluded from the movie. (Statistical analysis performed with one-way ANOVA. *p*<0.05 was considered significant).
(TIF)

S4 Fig Evaluation of Crk expression in wild type and Crk-/- knockout cells. The cell lysates from wild-type and Crk^-/-^ cells were analyzed for their CrkI and CrkII protein contents (the two isoforms of Crk) by Western blotting. As expected, CrkI and CrkII proteins are not expressed in Crk^-/-^ cells but they are in wild-type cells.
(TIF)

S5 Fig Determination of total focal adhesion area and by staining intensity measurement. Using ImageJ, cells were outlined using the fluorescent image channel and saved in a selection manager (blue outline, p-p130Cas is shown as a representative marker. A background subtraction process was applied to the fluorescent channel before setting a threshold. Particle analysis was performed on the previously selected cell outline in order to measure the puncta number and size (intensity) for each cell. Representative images show how puncta numbers and sizes are reduced in response to infection or transfection with ExoT.
(TIF)

S6 Fig ExoT and ExoT/ADPRT reduce FAK and p130Cas staining per FA structure. The staining intensities of either p-p130Cas or p-FAK per FA puncta was assessed, as described in S5 Fig, for cells either infected as indicated (A and B) or transfected as indicated (C and D). (Statistical analysis was performed using one-way ANOVA). Data indicate that ExoT and ExoT/ADPRT significantly reduce the recruitment of FAK and p130Cas to FA sites.
(TIF)

S7 Fig CrkI presence in FA sites in does not affect FA site structure or function in CrkI complemented Crk^-/-^ cells. Crk^-/-^ cells were transiently transfected with pIRES2-GFP expression vector harboring wild-type CrkI (pCrkI), or empty vector (pGFP). The impact of CrkI presence at FA sites (Fig 8) on FA structure and function was evaluated by determining the total number of FA puncta per cell (A), the ability of the cells to spread as determined by their surface area (B), and the ability of the cells to migrate as determined by the distance travelled within 24 hr (C). these data indicated that CrkI presence in FA did not affect FA structure and function in CrkI-complemented Crk^-/-^ cells (Student’s t-test, n = 50).
(TIF)

S8 Fig ExoT and ExoT/ADPRT disrupt localization of activated JNK to FA sites. HeLa cells were transfected with expression vectors harboring wild type ExoT (pExoT), ExoT with functional ADPRT domain (pExoT(G^-^A^+^)), empty vector (pGFP) or left untransfected. ~24 hr after transfection, cells were fixed and analyzed for phospho-JNK (p-JNK) by IF microscopy and representative images are shown.
(TIF)

S1 Table Strains and plasmids used in this study. (DOCX)

S1 Movie ExoT-intoxicated HeLa cells display substantial movement prior to their death. HeLa cells were infected with PA103Δ*exoU* (ΔU) at MOI ~10. Video images were captured every 15 min at 200× magnification. Cellular uptake of propidium iodide (PI), an impermeant nuclear dye, identifies dying cells (red). Note that following cell rounding the ExoT-intoxicated cell moves around the substrate preceding its death.
(MP4)

S2 Movie HeLa cells are susceptible to ExoT and ADPRT mediated cytotoxicity. HeLa cells were infected with either PA103Δ*exoU* (ΔU), PA103∆*exoU/exoT(R149K)* (∆U/T(G^-^A^+^)), or the T3SS mutant PA103 *pscJ*::*Tn5* (*pscJ*) at MOI ~10 or given PBS (Mock). Video images were captured every 15 min at 200× magnification. Cellular uptake of propidium iodide (PI), an impermeant nuclear dye, identifies dying cells (red). Note cytotoxicity caused by ΔU and ∆U/T(G^-^A^+^).
(MOV)

S3 Movie HeLa S3 cells are resistant to ExoT and ADPRT mediated cytotoxicity. HeLa S3 cells were infected with either PA103Δ*exoU* (ΔU), PA103∆*exoU/exoT(R149K)* (∆U/T(G^-^A^+^)), or the T3SS mutant PA103 *pscJ*::*Tn5* (*pscJ*) at MOI ~10 or given PBS (Mock). Video images were captured every 15 min at 200× magnification. Cellular uptake of propidium iodide (PI), an impermeant nuclear dye, identifies dying cells (red). Note the lack of cytotoxicity caused by ΔU and ∆U/T(G^-^A^+^).
(MOV)

S4 Movie Transient transfection with pCrkI/R38K is sufficient to cause cell death in HeLa cells that is apoptotic in nature. HeLa cells were transiently transfected with either pCrkI-GFP or the SH2 DN pCrkI/R38K-GFP expression vector or pCrkI/R38K-GFP pre-incubated with ZVAD, a broad pan-caspase inhibitor for 2h prior to transfection. Video images were captured every 15 min at 200× magnification. Cellular uptake of propidium iodide, an impermeant nuclear dye, identifies dying cells (red). Note that ZVAD significantly inhibits cell death of transfected cells.
(MOV)

S5 Movie Crk^-/-^ cells, complemented with GFP are resistant to ExoT-induced cytotoxicity. Crk^-/-^ cells were transfected with the pIRES2-GFP empty vector on consecutive days to increase transfection efficiency. 24 hr after final transfection, transfected cells were infected with PA103Δ*exoU* (ΔU), PA103∆*exoU/exoT(R149K)* (∆U/T(G^-^A^+^)), or the T3SS mutant PA103 *pscJ*::*Tn5* (*pscJ*) at MOI ~10. Video images were captured every 15 min at 200× magnification. Cellular uptake of propidium iodide (PI), an impermeant nuclear dye, identifies dying cells (red). For clarity, phase panels were excluded from the movie. Note that transfected cells (green) do not pick up PI.
(MP4)

S6 Movie Crk^-/-^ cells, complemented with CrkI, are susceptible to ExoT-induced cytotoxicity. Crk^-/-^ cells were transfected with the pCrkI-GFP vector on consecutive days to increase transfection efficiency. 24 hr after final transfection, transfected cells were infected with PA103Δ*exoU* (ΔU), PA103∆*exoU/exoT(R149K)* (∆U/T(G^-^A^+^)), or the T3SS mutant PA103 *pscJ*::*Tn5* (*pscJ*) at MOI ~10. Video images were captured every 15 min at 200× magnification. Cellular uptake of propidium iodide (PI), an impermeant nuclear dye, identifies dying cells (red). For clarity, phase panels were excluded from the movie. Note that transfected cells (green) do not pick up PI.
(MOV)

S7 Movie Transient transfection with pCrkI/R38K is sufficient to cause cytotoxicity in Crk^-/-^ cells. Crk^-/-^ cells were transfected with either pIRES2-GFP empty vector, pCrkI-GFP, pCrkI/R38K-GFP, or pCrkI/R38K,W170K-GFP. Video images were captured every 15 min at 200× magnification. Cellular uptake of propidium iodide, an impermeant nuclear dye, identifies dying cells (red). For clarity, phase panels were excluded from the movie. Note that only pCrkI/R38K transfected cells pick up PI.
(MOV)

S8 Movie The SH3 domain of CrkI must be functional for CrkI to mediate ExoT cytotoxicity. Crk^-/-^ cells were transfected with the pCrkI/R38K,W170K-GFP vector on consecutive days to increase transfection efficiency. 24 hr after final transfection, transfected cells were infected with PA103Δ*exoU* (ΔU), PA103∆*exoU/exoT(R149K)* (∆U/T(G^-^A^+^)), or the T3SS mutant PA103 *pscJ*::*Tn5* (*pscJ*) at MOI ~10. Video images were captured every 15 min at 200× magnification. Cellular uptake of propidium iodide (PI), an impermeant nuclear dye, identifies dying cells (red). For clarity, phase panels were excluded from the movie. Note that transfected cells (green) do not pick up PI
(MP4)
