# Supplementary figures and images for: Pseudomonas aeruginosa ExoT Induces Atypical Anoikis Apoptosis in Target Host Cells by Transforming Crk Adaptor Protein into a Cytotoxin
Source: PLoS Pathog. 2015 May 28;11(5):e1004934. doi: 10.1371/journal.ppat.1004934 (PMC4447348; doi:10.1371/journal.ppat.1004934)

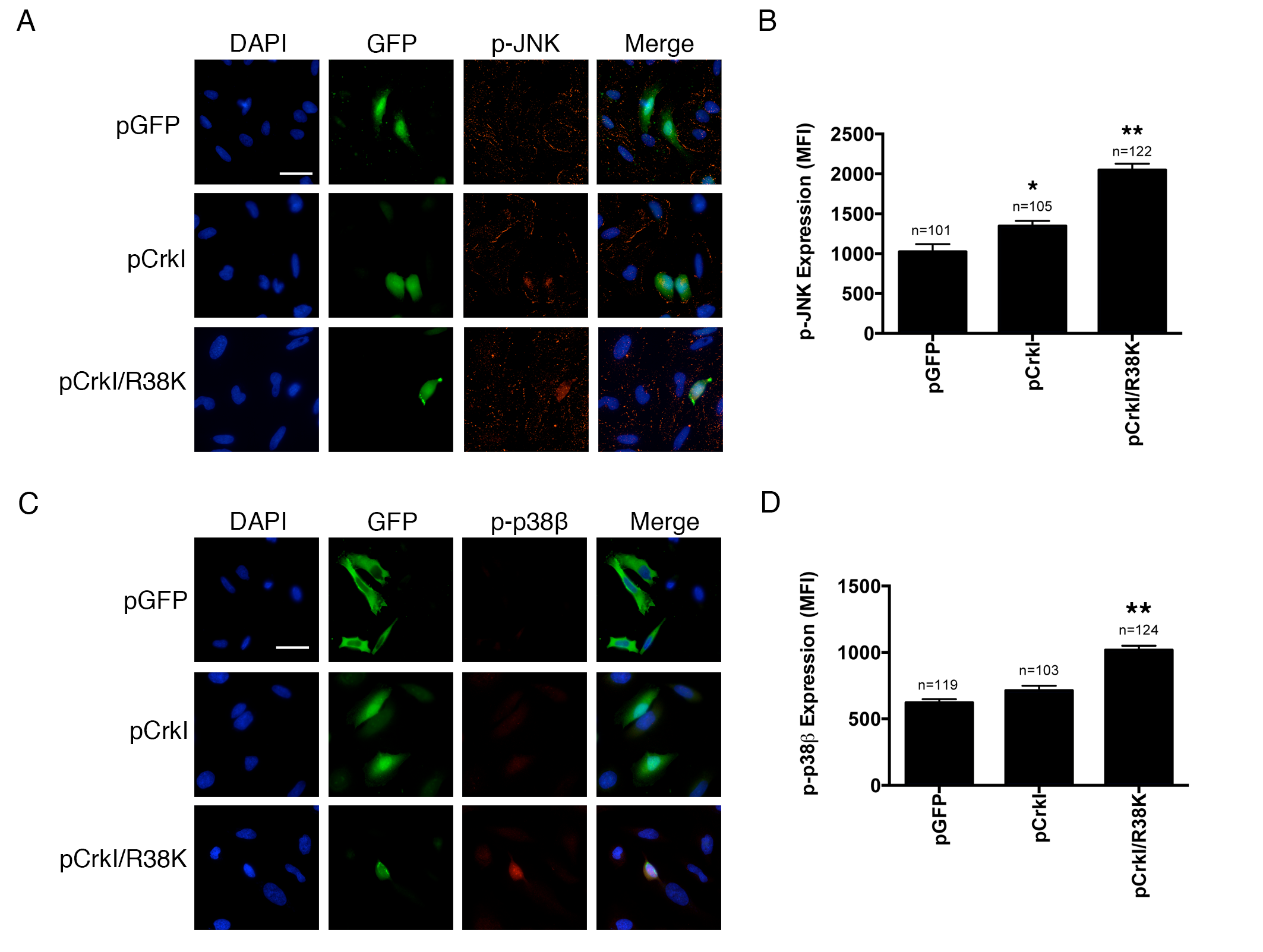

Supplement: S1 Fig — HeLa cells were transiently transfected with pIRES2-GFP expression vector harboring wild-type CrkI (pCrkI), SH2 DN (pCrkI/R38K), or empty vector (pGFP). 24 hr after transfection, cells were fixed and analyzed for p-JNK or p-p38β by IF microscopy. Representative images are shown in (A) and (C) and the expression levels were determined by densitometry and are shown as the mean fluorescent intensity (MFI) ± SEM in (B) and (D) respectively. (* Signifies p<0.05 and ** signifies p<0.001 by one-way ANOVA. Scale bar = 25μm). (TIF) [file ppat.1004934.s001.tif]

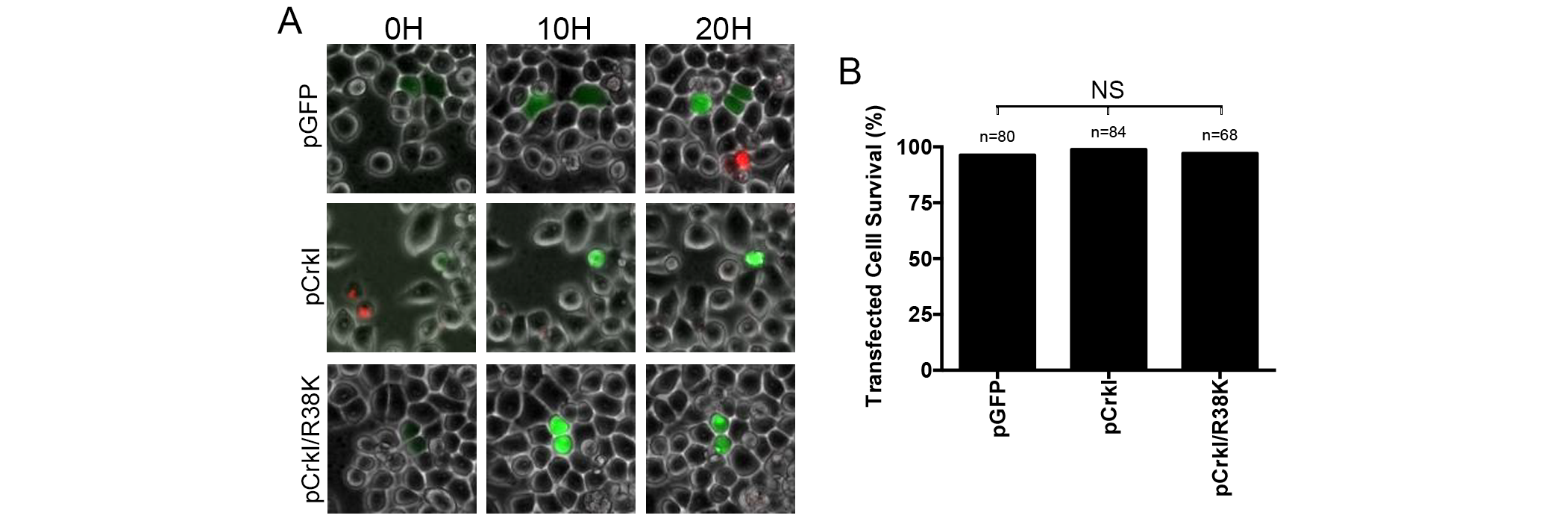

Supplement: S2 Fig — HeLa S3 cells were transiently transfected with pIRES2-GFP expression vector harboring ExoT (pExoT), ExoT/ADPRT (pExoT(G-A+), inactive ExoT (pExoT(G-A-), wild-type CrkI (pCrkI), or pCrkI/R38K mutant, all C-terminally fused to GFP, or empty vector (pGFP). Cytotoxicity of transfected host cells (green) was assessed by fluorescent time-lapse microscopy, using PI uptake, as the marker for cell death (red cells are dead). Video images were captured every 15 min and selected movie frames at indicated time points are shown in (A) and the corresponding data, expressed as a percentage of the total number of transfected cells, are shown in (B). Data was assessed by one-way ANOVA. (TIF) [file ppat.1004934.s002.tif]

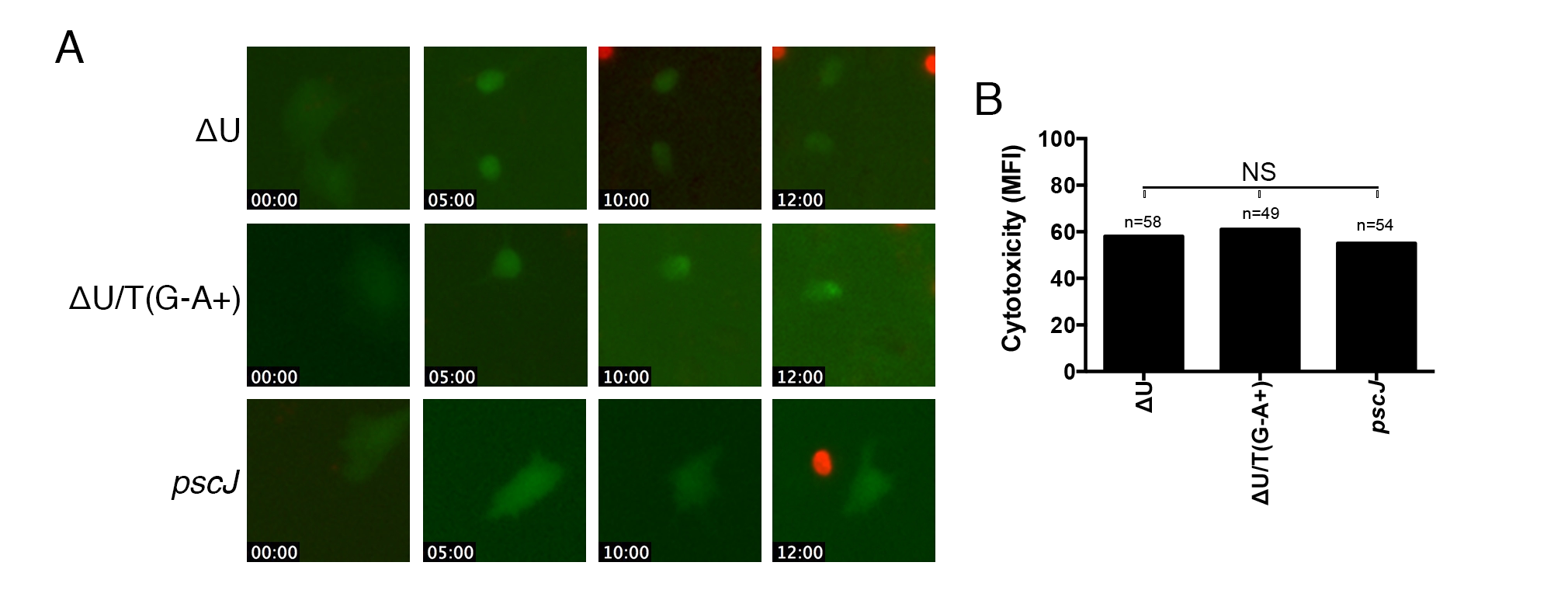

Supplement: S3 Fig — Crk-/- cells were transfected with the pCrkI/R38K,W170K-GFP vector, harboring null mutations in the SH2 and SH3 domains of CrkI, on consecutive days to increase transfection efficiency. 24 hr after final transfection, transfected cells were infected with PA103ΔexoU (ΔU), PA103∆exoU/exoT(R149K) (∆U/T(G-A+)), or the T3SS mutant PA103 pscJ::Tn5 (pscJ) at MOI ~10. Cytotoxicity of transfected host cells (green) was assessed by fluorescent time-lapse microscopy, using PI uptake, as the marker for cell death (red cells are dead). Video images were captured every 15 min and selected movie frames at indicated time points are shown in (A) and the corresponding data, expressed as a percentage of the total number of transfected cells, are shown in (B). For clarity, phase panels were excluded from the movie. (Statistical analysis performed with one-way ANOVA. p<0.05 was considered significant). (TIF) [file ppat.1004934.s003.tif]

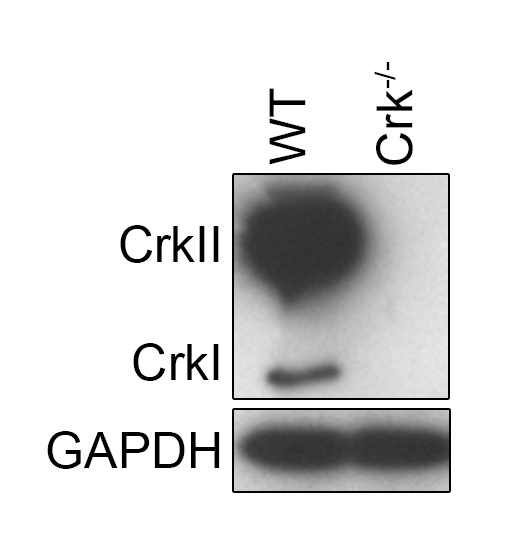

Supplement: S4 Fig — The cell lysates from wild-type and Crk-/- cells were analyzed for their CrkI and CrkII protein contents (the two isoforms of Crk) by Western blotting. As expected, CrkI and CrkII proteins are not expressed in Crk-/- cells but they are in wild-type cells. (TIF) [file ppat.1004934.s004.tif]

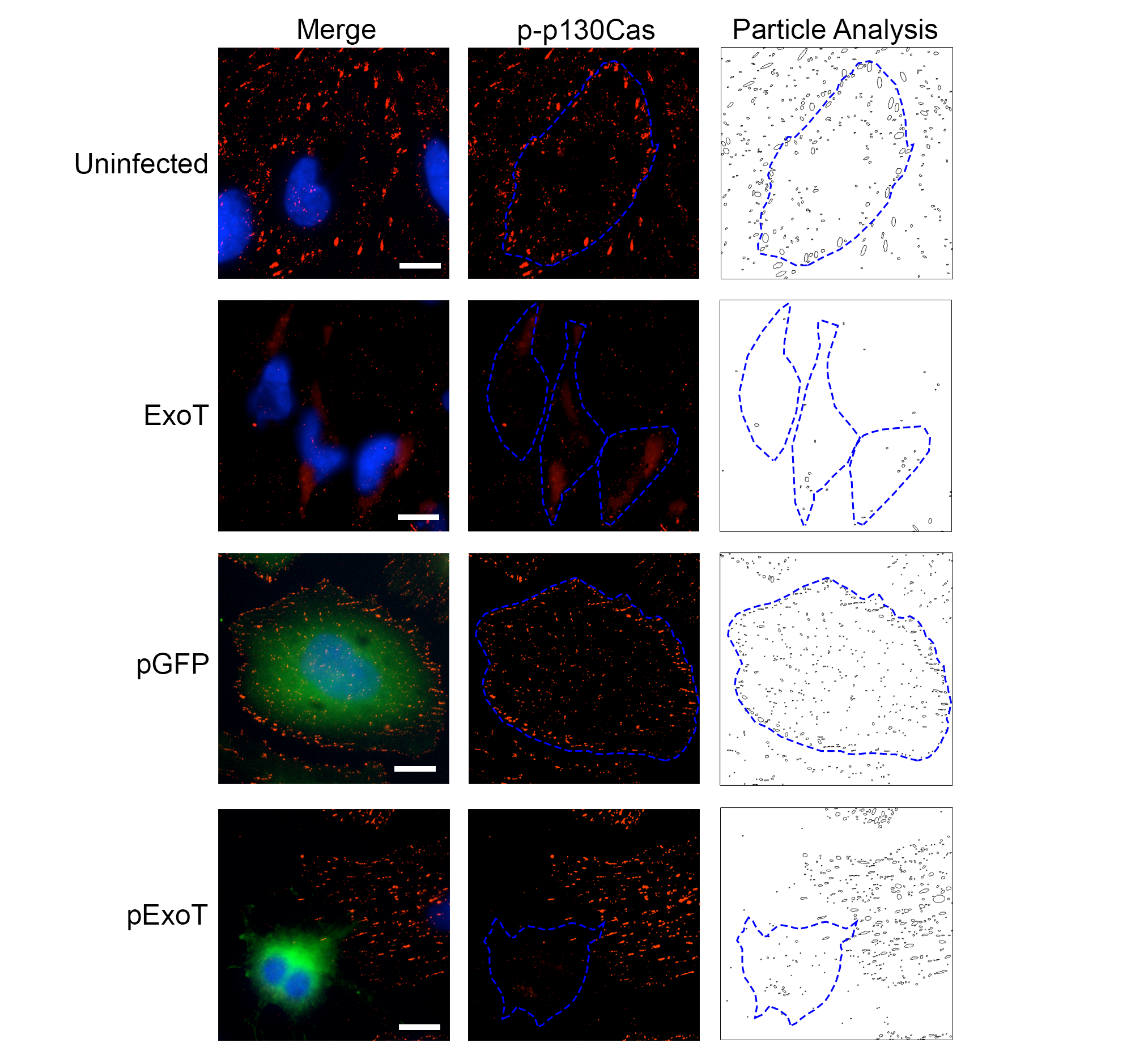

Supplement: S5 Fig — Using ImageJ, cells were outlined using the fluorescent image channel and saved in a selection manager (blue outline, p-p130Cas is shown as a representative marker. A background subtraction process was applied to the fluorescent channel before setting a threshold. Particle analysis was performed on the previously selected cell outline in order to measure the puncta number and size (intensity) for each cell. Representative images show how puncta numbers and sizes are reduced in response to infection or transfection with ExoT. (TIF) [file ppat.1004934.s005.tif]

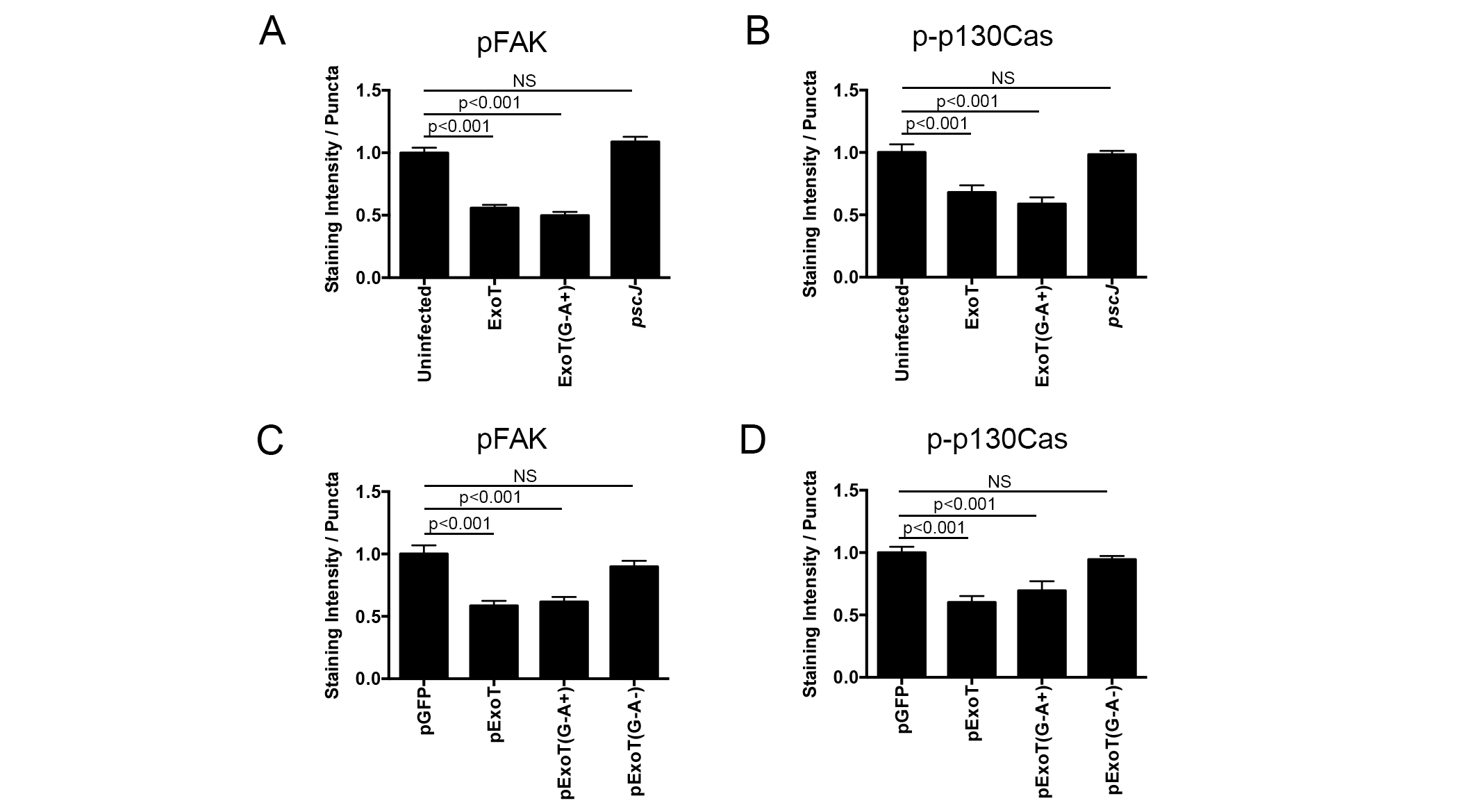

Supplement: S6 Fig — The staining intensities of either p-p130Cas or p-FAK per FA puncta was assessed, as described in S5 Fig, for cells either infected as indicated (A and B) or transfected as indicated (C and D). (Statistical analysis was performed using one-way ANOVA). Data indicate that ExoT and ExoT/ADPRT significantly reduce the recruitment of FAK and p130Cas to FA sites. (TIF) [file ppat.1004934.s006.tif]

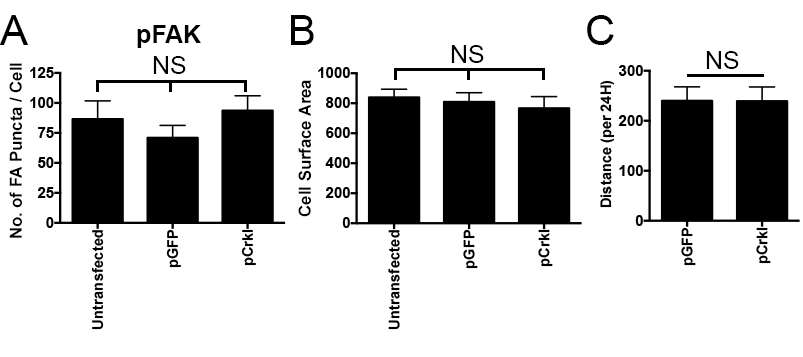

Supplement: S7 Fig — Crk-/- cells were transiently transfected with pIRES2-GFP expression vector harboring wild-type CrkI (pCrkI), or empty vector (pGFP). The impact of CrkI presence at FA sites (Fig 8) on FA structure and function was evaluated by determining the total number of FA puncta per cell (A), the ability of the cells to spread as determined by their surface area (B), and the ability of the cells to migrate as determined by the distance travelled within 24 hr (C). these data indicated that CrkI presence in FA did not affect FA structure and function in CrkI-complemented Crk-/- cells (Student’s t-test, n = 50). (TIF) [file ppat.1004934.s007.tif]

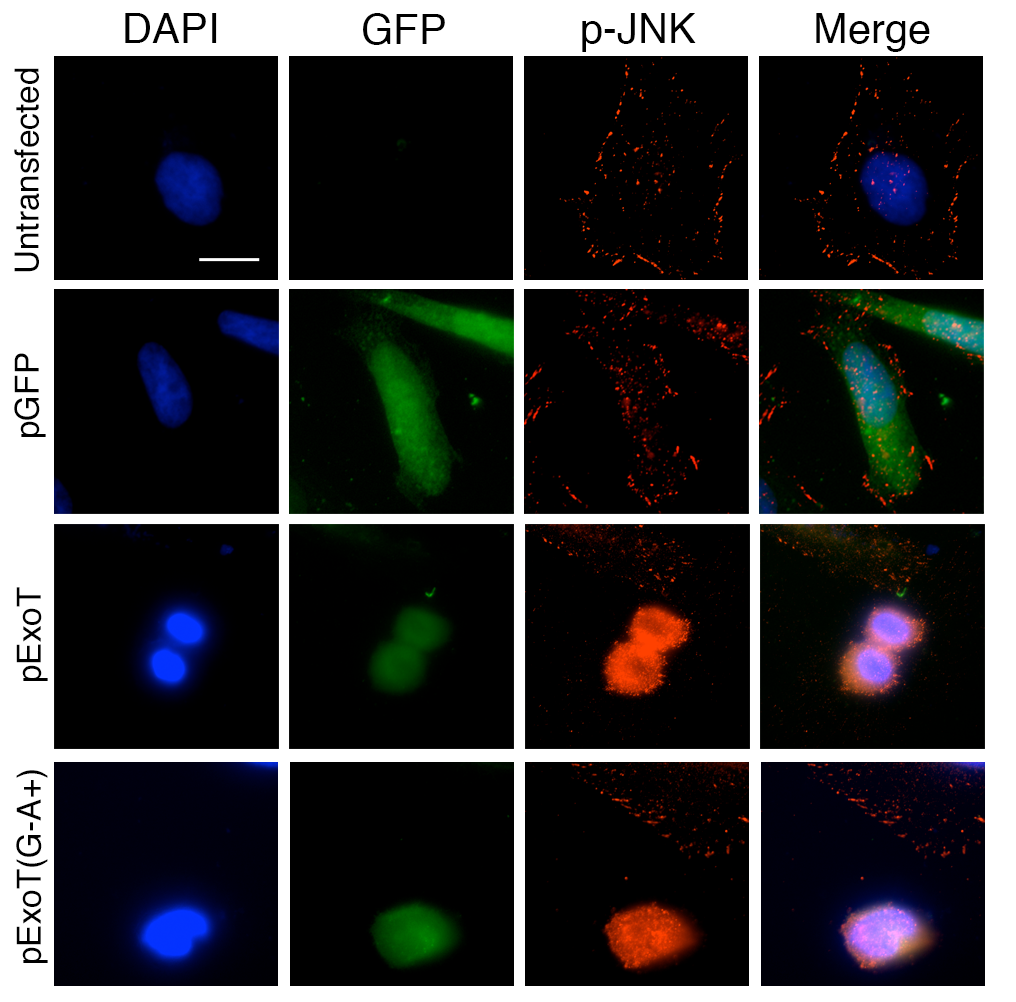

Supplement: S8 Fig — HeLa cells were transfected with expression vectors harboring wild type ExoT (pExoT), ExoT with functional ADPRT domain (pExoT(G-A+)), empty vector (pGFP) or left untransfected. ~24 hr after transfection, cells were fixed and analyzed for phospho-JNK (p-JNK) by IF microscopy and representative images are shown. (TIF) [file ppat.1004934.s008.tif]
