# Supplementary material for: Pseudomonas aeruginosa ExoT Induces Atypical Anoikis Apoptosis in Target Host Cells by Transforming Crk Adaptor Protein into a Cytotoxin
Source: PLoS Pathog. 2015 May 28;11(5):e1004934. doi: 10.1371/journal.ppat.1004934 (PMC4447348; doi:10.1371/journal.ppat.1004934)
Supplement: S1 Table — (DOCX) [file ppat.1004934.s009.docx]

**Table S1. Strains and plasmids used in this study.**

| **Strains or plasmids** | **Abbreviated Name** | **Relevant Characteristics** | **Reference or Source** |
| --- | --- | --- | --- |
| **Strains** |  |  |  |
| PA103Δ*exoU* | ∆U | *P. aeruginosa* expressing ExoT with an inframe deletion of ExoU | [1] |
| PA103Δ*exoU*/*exoT(R149K)* | ∆U/T(G^-^A^+^) | PA103ΔU with a point mutation in the GAP domain of ExoT | [2] |
| PA103Δ*exoU/exoT(R149K/AAA)* | ∆U/T(G^-^A^-^) | PA103ΔU with a point mutation in the GAP (R149K) and ADPRT (EQE383-385AAA) domains of ExoT | [3] |
| PA103*pscJ*::*Tn5* | *pscJ* | Tn Gent^r^ inserted into *pscJ*. T3SS defective | [4] |
| **Plasmids** |  |  |  |
| pGFP | pGFP | pIRESK11-EGFP (Clonetech) with bases 1870-1910 removed | [5] |
| pExoT-GFP | pExoT | pIRES2-EGFP harboring ExoT directly fused at its C-terminus to EGFP | [5] |
| pExoT(G^-^A^+^)-GFP | pExoT(G^-^A^+^) | pIRES2-EGFP harboring ExoT with a mutant GAP domain and a functional ADPRT domain, directly fused at its C-terminus to EGFP | [5] |
| pExoT(G^-^A^-^)-GFP | pExoT(G^-^A^-^) | pIRES2-EGFP harboring ExoT double mutant directly fused at its C-terminus to EGFP | [5] |
| pCrkI-GFP | pCrkI | pIRES2-EGFP harboring c-CrkI directly fused at its C-terminus to EGFP | [6] |
| pCrkI/R38K-GFP | pCrkI/R38K | pIRES2-EGFP harboring CrkI with a SH2 domain DN mutation (R38K) | [6] |
| TOPFlash | TOPFlash | Luciferase reporter plasmid with a promoter that requires TCF binding | Upstate |

1. Garrity-Ryan L, Kazmierczak B, Kowal R, Commolli J, Hauser A, et al. (2000) The arginine finger domain of ExoT is required for actin cytoskeleton disruption and inhibition of internalization of *Pseudomonas aeruginosa* by epithelial cells and macrophages. Infect Immun 68: 7100-7113.

2. Geiser T, Kazmierczak B, Garrity-Ryan L, Matthay M, Engel J (2001) *Pseudomonas aeruginosa* ExoT inhibits in vitro lung epithelial wound repair. Cell Microbiol 3: 223-236.

3. Garrity-Ryan L, Shafikhani S, Balachandran P, Nguyen L, Oza J, et al. (2004) The ADP ribosyltransferase domain of *Pseudomonas aeruginosa* ExoT contributes to its biological activities. Infection and immunity 72: 546-558.

4. Kang PJ, Hauser AR, Apodaca G, Fleiszig SM, Wiener-Kronish J, et al. (1997) Identification of *Pseudomonas aeruginosa* genes required for epithelial cell injury. Mol Microbiol 24: 1249-1262.

5. Shafikhani SH, Engel J (2006) *Pseudomonas aeruginosa* type III-secreted toxin ExoT inhibits host-cell division by targeting cytokinesis at multiple steps. Proceedings of the National Academy of Sciences of the United States of America 103: 15605-15610.

6. Shafikhani SH, Mostov K, Engel J (2008) Focal adhesion components are essential for mammalian cell cytokinesis. Cell Cycle 7: 2868-2876.
